# Clinical Utility of Plasma Microbial Cell-Free DNA Sequencing Among Immunocompromised Patients With Pneumonia

**DOI:** 10.1093/ofid/ofae425

**Published:** 2024-07-22

**Authors:** Deng B Madut, Roy F Chemaly, Sanjeet S Dadwal, Joshua A Hill, Yeon Joo Lee, Ghady Haidar, Alfred Luk, Alexander Drelick, Peter V Chin-Hong, Esther Benamu, Fareed Khawaja, Deepa Nanayakkara, Genovefa A Papanicolaou, Catherine Butkus Small, Monica Fung, Michelle Barron, Thomas Davis, Micah T McClain, Eileen K Maziarz, Armando D Bedoya, Daniel L Gilstrap, Jamie L Todd, Christina E Barkauskas, Madeleine R Heldman, Robert Bigelow, Jeffrey D Leimberger, Ephraim L Tsalik, Olivia Wolf, Mona Mughar, Constance Lau, Nicholas Noll, Desiree Hollemon, Radha Duttagupta, Daniel S Lupu, Sivan Bercovici, Bradley A Perkins, Timothy A Blauwkamp, Vance G Fowler, Thomas L Holland, Stephen P Bergin

**Affiliations:** Duke University School of Medicine, Durham, North Carolina, USA; The University of Texas MD Anderson Cancer Center, Houston, Texas, USA; City of Hope National Medical Center, Duarte California, USA; Fred Hutchinson Cancer Center, Seattle, Washington, USA; University of Washington School of Medicine, Seattle, Washington, USA; Memorial Sloan Kettering Cancer Center, New York, New York, USA; Weill Cornell Medicine, New York, New York, USA; University of Pittsburgh Medical Center, Pittsburgh, Pennsylvania, USA; Tulane University School of Medicine, New Orleans, Louisiana, USA; Weill Cornell Medicine, New York, New York, USA; New York-Presbyterian Hospital, New York, New York, USA; University of California San Francisco, San Francisco, California, USA; University of Colorado, Aurora, Colorado, USA; The University of Texas MD Anderson Cancer Center, Houston, Texas, USA; City of Hope National Medical Center, Duarte California, USA; Memorial Sloan Kettering Cancer Center, New York, New York, USA; Weill Cornell Medicine, New York, New York, USA; Weill Cornell Medicine, New York, New York, USA; New York-Presbyterian Hospital, New York, New York, USA; University of California San Francisco, San Francisco, California, USA; University of Colorado, Aurora, Colorado, USA; Indiana University School of Medicine, Indianapolis Indiana, USA; Duke University School of Medicine, Durham, North Carolina, USA; Duke Clinical Research Institute, Durham, North Carolina, USA; Duke University School of Medicine, Durham, North Carolina, USA; Duke University School of Medicine, Durham, North Carolina, USA; Duke University School of Medicine, Durham, North Carolina, USA; Duke University School of Medicine, Durham, North Carolina, USA; Duke Clinical Research Institute, Durham, North Carolina, USA; Duke University School of Medicine, Durham, North Carolina, USA; Duke University School of Medicine, Durham, North Carolina, USA; Duke Clinical Research Institute, Durham, North Carolina, USA; Duke Clinical Research Institute, Durham, North Carolina, USA; Duke University School of Medicine, Durham, North Carolina, USA; Durham Veterans Affairs Health Care System, Durham, North Carolina, USA; Danaher Diagnostics, Washington, DC, USA; Duke Clinical Research Institute, Durham, North Carolina, USA; Karius Inc., Redwood City, California, USA; Karius Inc., Redwood City, California, USA; Karius Inc., Redwood City, California, USA; Karius Inc., Redwood City, California, USA; Karius Inc., Redwood City, California, USA; Karius Inc., Redwood City, California, USA; Karius Inc., Redwood City, California, USA; Karius Inc., Redwood City, California, USA; Karius Inc., Redwood City, California, USA; Duke University School of Medicine, Durham, North Carolina, USA; Duke Clinical Research Institute, Durham, North Carolina, USA; Duke University School of Medicine, Durham, North Carolina, USA; Duke Clinical Research Institute, Durham, North Carolina, USA; Duke University School of Medicine, Durham, North Carolina, USA; Duke Clinical Research Institute, Durham, North Carolina, USA

**Keywords:** immunocompromised, microbial cell-free DNA sequencing, pneumonia

## Abstract

**Background:**

Plasma microbial cell-free DNA (mcfDNA) sequencing can establish the etiology of multiple infectious syndromes by identifying microbial DNA in plasma. However, data are needed to define the clinical scenarios where this tool offers the highest clinical benefit.

**Methods:**

We conducted a prospective multicenter observational study that evaluated the impact of plasma mcfDNA sequencing compared with usual care testing among adults with hematologic malignancies. This is a secondary analysis of an expanded cohort that evaluated the clinical utility of plasma mcfDNA sequencing across prespecified and adjudicated outcomes. We examined the percentage of participants for whom plasma mcfDNA sequencing identified a probable cause of pneumonia or clinically relevant nonpneumonia infection. We then assessed potential changes in antimicrobial therapy based on plasma mcfDNA sequencing results and the potential for early mcfDNA testing to avoid bronchoscopy and its associated adverse events.

**Results:**

Of 223 participants, at least 1 microbial detection by plasma mcfDNA sequencing was adjudicated as a probable cause of pneumonia in 57 (25.6%) and a clinically relevant nonpneumonia infection in 88 (39.5%). A probable cause of pneumonia was exclusively identified by plasma mcfDNA sequencing in 23 (10.3%) participants. Antimicrobial therapy would have changed for 41 (18.4%) participants had plasma mcfDNA results been available in real time. Among the 57 participants with a probable cause of pneumonia identified by plasma mcfDNA sequencing, bronchoscopy identified no additional probable cause of pneumonia in 52 (91.2%).

**Conclusions:**

Plasma mcfDNA sequencing could improve management of both pneumonia and other concurrent infections in immunocompromised patients with suspected pneumonia.

Pneumonia is a common life-threatening infection among patients with hematologic malignancies [1–3]. There is no microbiologic gold standard to establish an etiology of pneumonia, and all routinely used tests have limitations [4]. For example, respiratory cultures are constrained by slow turnaround times and low sensitivity, particularly for fastidious organisms. Molecular methods such as nucleic acid amplification tests can overcome many of the constraints of culture but are limited by the number of pathogens that can be incorporated into multiplex assays. Given the susceptibility of patients with hematologic malignancies to a wide range of pneumonia-causing pathogens and the impact of timely diagnosis on outcomes [5], it is imperative to identify novel techniques that enhance existing diagnostic yield.

Plasma microbial cell-free DNA (mcfDNA) sequencing has emerged as a promising tool for the diagnosis and management of infectious diseases [6]. Compared with conventional microbiologic tests, plasma mcfDNA sequencing offers several advantages including short turnaround times and unbiased detection of a wide range of pathogens. Increasing evidence suggests that plasma mcfDNA sequencing may be particularly valuable when applied to immunocompromised hosts [7–10]. Specifically, studies suggest that microbial detections by plasma mcfDNA sequencing may have a positive impact on clinical management for immunocompromised patients, including those with deep-seated infections such as pneumonia [8–10]. However, prospective studies are required to clearly define the magnitude of these benefits in this patient population.

In the prospective observational Pneumonia in the Immunocompromised—Use of the Karius test for the Detection of Undiagnosed Pathogens (PICKUP) study, Bergin et al. evaluated the additive diagnostic value of plasma mcfDNA sequencing compared with conventional microbiologic diagnoses for suspected pneumonia among patients with hematologic malignancies [11]. The addition of plasma mcfDNA sequencing to conventional microbiologic diagnostics increased the diagnostic yield for pneumonia by 12%. Presented here is a secondary analysis of an expanded PICKUP cohort that aims to further define the clinical utility of plasma mcfDNA sequencing in this patient population.

## METHODS

### Study Design

The PICKUP study was a prospective observational study conducted at 10 tertiary care centers in the United States (ClinicalTrials.gov: NCT04047719) [11]. Hospitalized adults were eligible for enrollment if they were undergoing a diagnostic bronchoscopy to establish a microbiologic etiology of pneumonia and met at least 1 of the following criteria: (1) received chemotherapy for treatment of hematologic malignancy in the past 45 days; (2) had relapsed hematologic malignancy with chemotherapy anticipated in the next 45 days; (3) underwent recent hematopoietic cell transplantation; or (4) were receiving immunosuppressive therapy for active graft-vs-host disease. Patients with an established microbiologic etiology of pneumonia before screening or any positive molecular test for severe acute respiratory syndrome coronavirus 2 in the previous 14 days were excluded. Eligible patients underwent bronchoscopy no more than 1 day before enrollment or were scheduled for bronchoscopy within 5 days of enrollment.

### Study Procedures

Clinician documentation and results of all imaging and microbiologic, molecular, and serologic testing performed per usual care from 3 days before through 14 days after enrollment were recorded. In addition, participants were followed for up to 50 days to capture the final culture or reference laboratory testing results, key clinical outcomes, and antimicrobial exposure through study completion. The study protocol specified a stringent minimum diagnostic evaluation to establish the etiology of pneumonia for all participants. To adhere to the minimum diagnostic evaluation, blood and nasopharyngeal swab samples were collected within 24 hours of enrollment. Noninvasive tests required to complete the minimum diagnostic evaluation included blood culture, serum galactomannan, and a multiplex polymerase chain reaction (PCR) respiratory viral panel on nasopharyngeal samples. The minimum diagnostic evaluation from bronchoalveolar lavage (BAL) fluid included bacterial, fungal, and mycobacterial cultures, as well as staining or molecular testing for *Pneumocystis jirovecii*. If the minimum diagnostic evaluation from BAL fluid was not met by usual care testing, archived BAL fluid was sent for supplemental testing at a centralized reference laboratory (Indiana University Core Laboratory). Participants who completed the protocol-required usual care testing were classified as the per-protocol population. Primary findings of the per-protocol population have been published [11]. Presented here is an expanded analysis of all participants who had an adjudicated plasma mcfDNA sequencing test.

### Plasma Microbial Cell-Free DNA Sequencing

Blood samples for mcfDNA sequencing were collected on days 1, 3, or 4 and 5 or 6 after enrollment and processed into plasma. Plasma samples were shipped to the Clinical Laboratory Improvements Amendments–certified and College of American Pathologists–accredited Karius Laboratory (Redwood City, CA, USA) to detect mcfDNA. Test methods and validation have been previously described [12]. Briefly, microbial taxa abundances were estimated using a database of >20 000 curated assemblies from >16 000 species. All mcfDNA concentrations from organisms present above a statistical threshold were reported by the Karius, version 3.6 and 3.7, analytical pipeline in molecules per microliter (MPM). An example of the Karius test report provided to adjudication committee members is available in the Supplementary Data. Results of plasma mcfDNA sequencing were not provided to members of the clinical team caring for enrolled patients.

### Adjudication Process

A committee of 4 infectious diseases (D.M., E.M., M.M., T.H.) and 4 pulmonary medicine (A.B., C.B., D.G., J.T.) physicians with clinical expertise caring for immunocompromised patients with pneumonia adjudicated all cases in a 2-step process. Adjudicators, blinded to the plasma mcfDNA sequencing report, first reviewed a composite of all clinical documentation and usual care diagnostic testing results to determine if a probable cause of the index pneumonia event was identified within 14 days of study enrollment. Following review and adjudication of usual care testing, results of plasma mcfDNA sequencing collected on day 1 of study enrollment were then unblinded and adjudicated. Adjudicators classified each microbe identified by plasma mcfDNA sequencing as (1) a probable cause of the index pneumonia event, (2) a probable cause of a clinically relevant nonpneumonia infection, or (3) not causing an active infection (commensal organism or contaminant). When a microbe was identified as a probable cause of pneumonia or a clinically relevant nonpneumonia infection, adjudicators determined whether the diagnostic information would have changed antimicrobial management if the results were available in real time. Disagreements between primary adjudicators were resolved by a committee comprised of at least 3 adjudicators.

For each probable cause of pneumonia identified by usual care, 2 reviewers (D.M. and S.B.) determined the first point in time (measured in days) for which that test result would have yielded sufficient diagnostic information to impact antimicrobial management by members of the treatment team. This point was used to analyze the median time to clinically actionable results for each usual care result. If a disagreement occurred between the 2 reviewers, a third reviewer (T.H.) resolved the disagreement.

### Study Objectives and Definitions

All objectives were specified in the study protocol before the enrollment of participants. This secondary analysis examines the clinical utility of plasma mcfDNA sequencing across 5 outcomes. First, the additive diagnostic value of plasma mcfDNA sequencing for pneumonia was compared with noninvasive and invasive usual care testing. Additive diagnostic value—previously reported in the per-protocol population [11]—was defined as the percentage of participants with a probable cause of pneumonia exclusively identified by plasma mcfDNA sequencing. Second, we examined the percentage of participants who could have avoided bronchoscopy and associated complications if plasma mcfDNA sequencing results were available in real time. The potential avoidance of bronchoscopy was considered in participants for whom plasma mcfDNA sequencing exclusively identified a probable cause of pneumonia (additive diagnostic value) or identified the same probable cause of pneumonia as bronchoscopy. Third, we examined the time in days to a clinically actionable result for plasma mcfDNA sequencing compared with usual care testing—defined as the protocol-required minimum diagnostic evaluation plus any additional diagnostic testing performed during routine care to establish a pathogenic cause of pneumonia. Fourth, we examined the percentage of participants for whom plasma mcfDNA sequencing identified at least 1 pathogen that was adjudicated as a clinically relevant nonpneumonia infection. Finally, we evaluated the potential impact of plasma mcfDNA sequencing on antimicrobial prescribing for pneumonia and clinically relevant nonpneumonia infections if results were available to treating clinicians in real time.

### Statistical Analysis

Data analysis was performed using SAS, version 9.4 (SAS Institute, Cary, NC, USA). All study participants with an adjudicated day 1 plasma mcfDNA sequencing result were included. Continuous variables were expressed using medians and interquartile ranges (IQRs). Categorical variables were expressed as frequencies. The cumulative incidence of a probable cause of pneumonia identified by usual care or plasma mcfDNA sequencing was estimated using the Kaplan-Meier method with censoring at 14 days after enrollment. The median (IQR) clinically actionable time point in days was calculated for the first usual care test that identified a probable cause of pneumonia. Both the cumulative incidence and clinically actionable time point analyses assumed a median (IQR) time in days from sample collection to results of 2.63 (2.04–3.71) days for plasma mcfDNA sequencing based on published data from >15 000 real-world tests [13].

## RESULTS

### Demographic and Clinical Characteristics

From January 3, 2020, through February 4, 2022, 257 participants were enrolled at 10 US medical centers, and 223 (86.8%) were included in this analysis (Supplementary Figure 1). Of participants analyzed, the median (IQR) age was 62 (50–69) years, 72 (32.3%) were female, 169 (75.8%) received chemotherapy within 45 days of enrollment, 127 (57.0%) had relapsed disease at the time of enrollment, 69 (30.9%) had received a hematopoietic cell transplant within the past year, and 23 (10.3%) were receiving immunosuppression for active graft-vs-host disease. Demographic and clinical characteristics are shown in Table 1.

**Table 1.** Characteristics of Participants Enrolled in the Pneumonia in the Immunocompromised—Use of the Karius Test for the Detection of Undiagnosed Pathogens (PICKUP) Study, 2020–2022

Characteristics	Overall^a^ (n = 223)
Age, median (IQR), y	62.0 (50.0–69.0)
Female	72 (32.3)
Leukemia	146 (65.5)
Lymphoma	42 (18.8)
Myelodysplastic syndromes	40 (17.9)
Multiple myeloma	21 (9.4)
Transplant	69 (30.9)
Autologous stem cell transplant	9 (13.0)
Allogenic stem cell transplant	59 (85.5)
Chemotherapy w/in 45 d of enrollment	169 (75.8)
Relapse at time of enrollment	127 (57.0)
Remission at time of enrollment	37 (16.6)
Active graft-vs-host disease	26 (11.7)
Immunosuppressive pharmacologic treatment	23 (10.3)
Invasive procedure 1 d before enrollment to day 14^b^	222 (99.6)
Bronchoscopy^c^	222 (100.0)
Thoracentesis	10 (4.5)
Transthoracic needle aspiration	3 (1.4)
Mini BAL	1 (0.5)
White blood cell count, median (IQR), K/µL	1.50 (0.40–4.20)
Absolute neutrophil count, median (IQR), K/µL	0.69 (0.04–3.04)
Anti-infective medication at enrollment	223 (100.0)
Antipseudomonal antibacterial	209 (93.7)
Mold-active antifungal	161 (72.2)
Anti-MRSA antibacterial	146 (65.5)
Anti-PJP antimicrobial	54 (24.2)
Death ≤30 d	37 (16.6)
Overall study mortality	54 (24.2)

Abbreviations: BAL, bronchoalveolar lavage; IQR, interquartile range; MRSA, methicillin-resistant *Staphylococcus aureus*; PJP, *Pneumocystis jiroveci* pneumonia.

^a^Data are presented as No. (%) unless otherwise indicated.

^b^Includes invasive diagnostic procedures performed to establish pneumonia etiology. Some patients underwent ≥1 diagnostic procedure. No video-assisted thoracoscopic surgeries were performed in this cohort.

^c^One study subject was scheduled for bronchoscopy but did not have the procedure due to declining health status.

### Additive Diagnostic Value for the Diagnosis of Pneumonia

**Figure 1.**
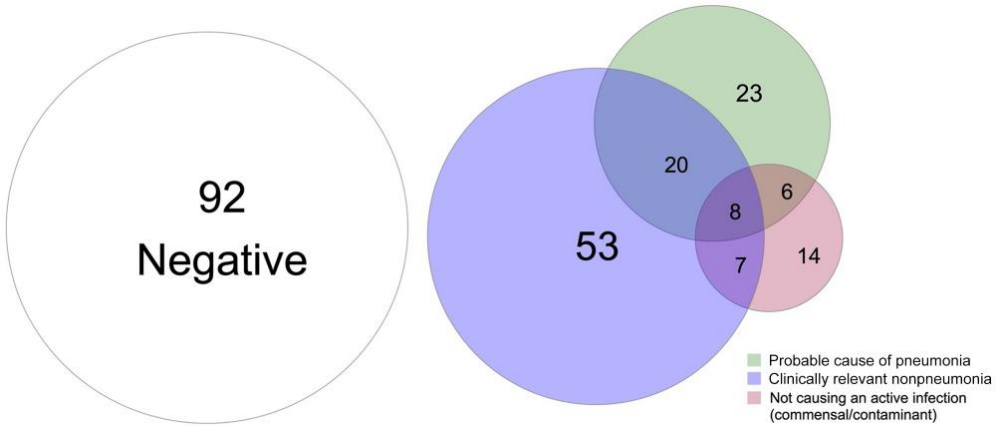
Proportional Venn diagram depicting adjudicated categories of plasma microbial cell-free DNA results among 223 participants enrolled in the Pneumonia in the Immunocompromised—Use of the Karius Test for the Detection of Undiagnosed Pathogens (PICKUP) study, 2020–2022. Of the 92 participants who did not have a microbe detected by plasma mcfDNA sequencing, a probable cause of pneumonia was identified by usual care in 21 (22.8%).

**Figure 2.**
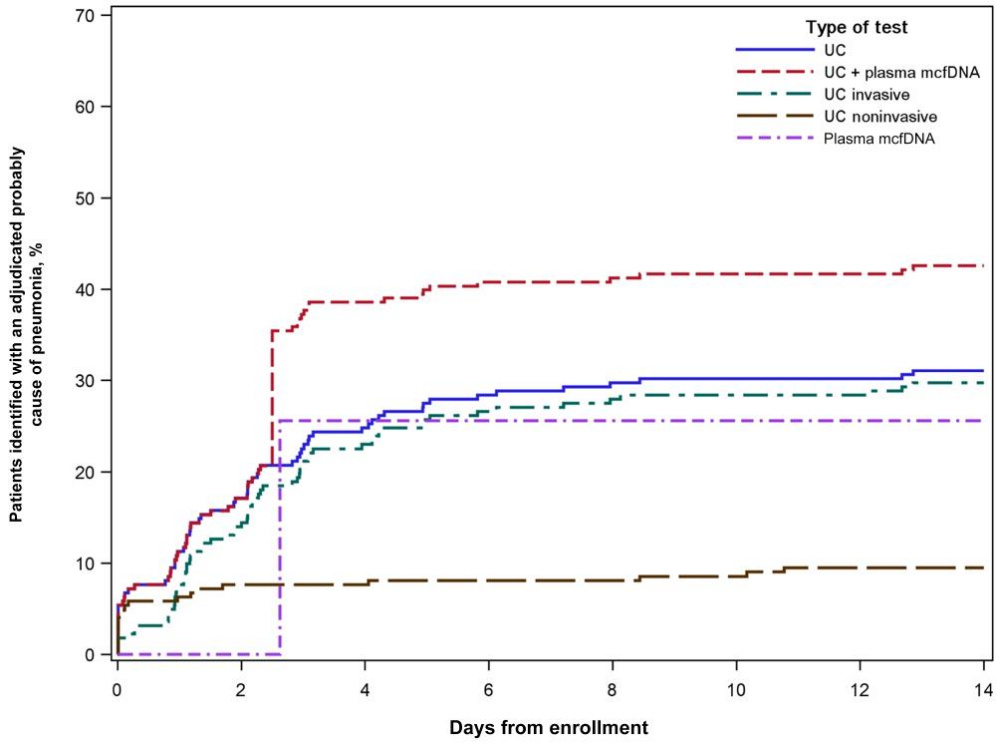
Incidence of adjudicated probable causes of pneumonia identified by usual care testing and/or plasma microbial cell-free DNA sequencing among participants enrolled in the Pneumonia in the Immunocompromised—Use of the Karius Test for the Detection of Undiagnosed Pathogens (PICKUP) study, 2020–2022. Plasma microbial cell-free DNA specimens were collected within 24 hours of enrollment. Some usual care specimens were collected before enrollment. Only tests with results reported within 14 days of enrollment were included.

Plasma mcfDNA sequencing identified at least 1 microbe in 131 (58.7%) participants, and at least 1 microbe was adjudicated as a probable cause of pneumonia in 57 (25.6%) (Figure 1; Supplementary Figures 2 and 3). Usual care testing identified a probable cause of pneumonia in 73 (32.7%) participants, and the combination of plasma mcfDNA sequencing and usual care identified a probable cause of pneumonia in 96 (43.0%) participants (Figure 2; Supplementary Figure 4). Of the 92 participants who did not have a microbe detected by plasma mcfDNA sequencing, a probable cause of pneumonia was identified by usual care in 21 (22.8%) (Figure 1).

**Figure 3.**
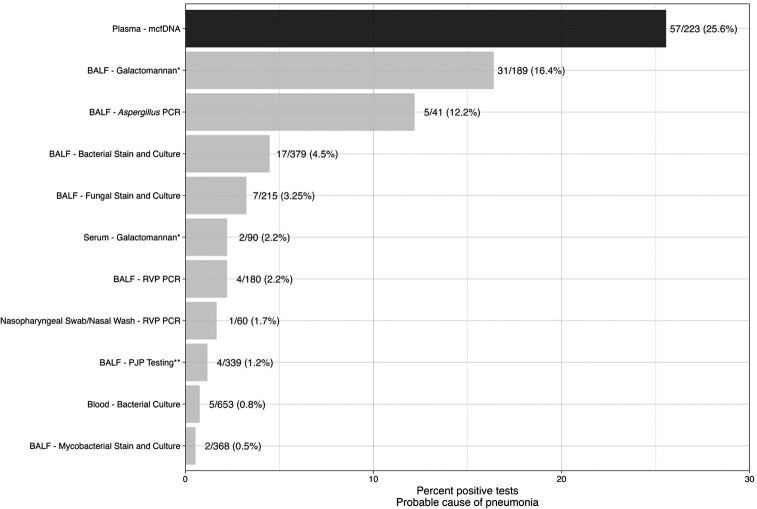
Identification of a probable cause of pneumonia for usual care testing and plasma microbial cell-free DNA sequencing among 223 participants enrolled in the Pneumonia in the Immunocompromised—Use of the Karius Test for the Detection of Undiagnosed Pathogens (PICKUP) study, 2020–2022. >1 BAL galactomannan was sent for some patients. A serum or BAL galactomannan >0.5 was categorized as a positive test. Includes stain and polymerase chain reaction. Abbreviations: BALF, bronchoalveolar lavage fluid; PCR, polymerase chain reaction; PJP, *Pneumocystis jiroveci* pneumonia; RVP, respiratory viral panel.

A probable cause of pneumonia was exclusively identified by plasma mcfDNA sequencing in 23 participants when compared with both invasive and noninvasive usual care testing—providing an overall additive diagnostic value of 10.3% (Supplementary Figure 5). Plasma mcfDNA sequencing exclusively identified the probable cause of pneumonia in 46 (20.6%) participants when compared with noninvasive usual care testing and in 24 (10.8%) when compared with invasive tests. The yield of identifying a probable cause of pneumonia observed for plasma mcfDNA sequencing (25.6%) was higher than any individual usual care test (Figure 3). Among all usual care tests collected via bronchoscopy, BAL galactomannan demonstrated the highest diagnostic yield (16.4%). The combined yield for all other usual care tests obtained by bronchoscopy was <24%. Usual care sputum testing was collected in a minority of participants and detected a pathogen in 4 of 67 (5.9%) specimens obtained (Supplementary Table 1).

### Inferred Avoidance of Bronchoscopy and Associated Complications

Bronchoscopy was performed on 222 participants. One participant was scheduled for a bronchoscopy but did not undergo the procedure because of clinical deterioration. Of the 57 participants with a probable cause of pneumonia identified by plasma mcfDNA sequencing, bronchoscopy identified no additional probable cause of pneumonia in 52 (91.2%). Among these 52 participants, plasma mcfDNA sequencing exclusively identified the probable cause of pneumonia in 25 (48.1%) and identified the same probable cause of pneumonia as bronchoscopy in 27 (51.9%). Bronchoscopy was associated with complications in 35 (15.8%) participants (Supplementary Table 2). Among participants with bronchoscopy-related complications, plasma mcfDNA sequencing exclusively identified the probable cause of pneumonia or identified the same probable cause of pneumonia as bronchoscopy in 11 (31.4%).

### Time to Clinically Actionable Result

The median (IQR) time to a clinically actionable result from enrollment for usual care tests that identified a probable cause of pneumonia was 1.5 (0.8–3.1) days (Supplementary Figure 6). For noninvasive usual care tests, the median time to a clinically actionable result (IQR) was 0.1 (0.0–1.3) days, and for samples collected by invasive means, the median time (IQR) was 2.1 (1.0–3.2) days. As highlighted previously, the median time to a clinically actionable result for plasma mcfDNA sequencing (IQR) was assumed at 2.63 (2.04–3.71) days [13].

### Clinically Relevant Nonpneumonia Infections

Among the 223 participants, plasma mcfDNA sequencing identified a clinically relevant nonpneumonia infection in 88 (39.5%) (Supplementary Figure 7). Collectively, plasma mcfDNA sequencing identified a probable cause of pneumonia or a clinically relevant nonpneumonia infection in 117 (52.5%) participants. In 14 (6.3%) participants, microbes detected by plasma microbial mcfDNA sequencing were adjudicated as not clinically relevant (Supplementary Table 3).

### Potential Impact of Plasma mcfDNA Sequencing on Antimicrobial Prescribing

The adjudication committee determined that plasma mcfDNA sequencing results would have changed antimicrobial therapy for pneumonia in 21 of 223 (9.4%) participants (Table 2; Supplementary Table 4). For the 23 participants for whom plasma mcfDNA sequencing exclusively identified a probable cause of pneumonia, antimicrobial therapy would have changed in 17 (73.9%). Plasma mcfDNA sequencing results would have changed antimicrobial therapy in 22 of 223 (9.9%) participants in whom a clinically relevant nonpneumonia infection was identified. Collectively, antimicrobial therapy would have changed for 41 (18.4%) participants if plasma mcfDNA sequencing results were available to treating clinicians.

**Table 2.** Adjudicated Changes in Antimicrobial Therapy for Participants Enrolled in the Pneumonia in the Immunocompromised—Use of the Karius Test for the Detection of Undiagnosed Pathogens (PICKUP) Study, 2020–2022

Antimicrobial Changes for All Patients With an Adjudicated Plasma Microbial Cell-Free DNA Test	Microbial Cell-Free DNA vs All Usual Care Microbiologic Testing for Pneumonia	Microbial Cell-Free DNA vs All Usual Care Microbiologic Testing for Clinically Relevant Nonpneumonia
Any antimicrobial therapy change, No. (%)	21/223 (9.4)	22/223 (9.9)
Potential antibacterial therapy changes, No. (%)	14/223 (6.3)	17/223 (7.6)
Broaden antibacterial coverage	7	13
Additional aerobic gram-negative coverage	1	1
Additional anaerobic coverage	3	5
Additional anaerobic coverage AND additional aerobic gram-negative coverage		2
Other	3^a^	5^b^
Narrow antibacterial coverage	7	
Stopped ≥1 gram-negative agent	2	
Stopped MRSA coverage	2	
Stopped MRSA coverage AND stopped ≥1 gram-negative agent	2	
Stopped MRSA coverage AND stopped ≥1 anaerobic agent	1	
Earlier antibacterial coverage	2	4
Potential antiviral therapy changes, No. (%)	1/223 (0.4)	4/223 (1.8)
Added ≥1 agent	1	4
Potential antifungal therapy changes, No. (%)	7/223 (3.1)	2/223 (0.9)
Added ≥1 agent	3	2
Added ≥1 agent AND stopped ≥1 agent	1	
Stopped ≥1 agent	3	

Abbreviation: MRSA, methicillin-resistant *Staphylococcus aureus.*

^a^One of 3 added coverage for *Nocardia*, 1/3 added coverage for atypical organisms, and 1/3 added trimethoprim-sulfamethoxazole for *Pneumocystis jirovecii*.

^b^Four of 5 added vancomycin-resistant enterococci coverage, and 1/5 added more targeted coverage of viridans group streptococci.

## DISCUSSION

In this prespecified secondary analysis of an expanded cohort of the PICKUP study, we found that plasma mcfDNA sequencing identified pathogens associated with pneumonia and concurrent nonpneumonia infections. The availability of plasma microbial sequencing results to treating clinicians in real time would have impacted antimicrobial management and may have reduced the need for bronchoscopy and incidence of procedural complications.

Our results confirm an additive diagnostic value of plasma mcfDNA sequencing in this expanded cohort of the PICKUP study [11]. The additive diagnostic value of plasma mcfDNA sequencing was highest compared with noninvasive testing. This finding suggests that plasma mcfDNA sequencing could be particularly impactful in immunocompromised patients with pneumonia who are unsuitable for invasive sampling. In addition, our results suggest that the added value of pursuing a bronchoscopy should be carefully considered in circumstances where plasma mcfDNA sequencing identifies a pathogen that is a probable cause of pneumonia. Specifically, we observed that bronchoscopy provided no additional probable cause of pneumonia in 52 of the 57 participants for whom plasma mcfDNA sequencing identified a probable cause of pneumonia. Although the PICKUP study was designed to evaluate the additive diagnostic value of plasma mcfDNA sequencing at the time of bronchoscopy, these findings suggest that plasma mcfDNA sequencing performed concurrently with initial noninvasive testing may improve diagnostic testing efficiency and reduce the need for subsequent invasive procedures (Supplementary Figure 8).

We observed that plasma mcfDNA sequencing results would have impacted antimicrobial therapy for 18.4% of participants if the results were available to treating clinicians. While all participants were suspected to have pneumonia, potential changes to antimicrobial therapy were in part driven by the identification of clinically relevant nonpneumonia infections. This result is not unexpected as immunocompromised patients may harbor multiple infections or present with abnormal pulmonary findings as a result of systemic infections outside the lungs [14]. Consequently, our finding highlights the distinct advantages that the unbiased approach of plasma mcfDNA sequencing offers for this patient population.

The optimal time point for integrating plasma mcfDNA sequencing into current testing algorithms for immunocompromised patients with pneumonia remains uncertain. In part, established clinical guidance for diagnosing pneumonia among immunocompromised patients is still emerging [4]. Our findings suggest that a potential workflow could entail the use of plasma mcfDNA sequencing when initial noninvasive tests yield negative results and bronchoscopy is under consideration. Given the low probability of achieving a microbiologic diagnosis with noninvasive sampling, including plasma mcfDNA sequencing earlier in the diagnostic workup for immunocompromised patients with pneumonia warrants consideration.

The strengths of our study include the prospective, multicenter design and blinded centralized committee adjudication for key outcomes. Despite these strengths, limitations must be acknowledged. First, participants were enrolled from 10 tertiary medical centers; thus, the results may underestimate the diagnostic efficiency of plasma mcfDNA sequencing in medical centers with delayed access to bronchoscopy or extensive microbiologic testing. Second, microbes detected by plasma mcfDNA sequencing in 10.7% of patients were adjudicated as not causing a clinically relevant infection. While this proportion is lower than that previously reported [15], routine use of plasma mcfDNA will require careful interpretation of results by physicians with expertise in treating infectious diseases. In addition, further studies are needed to quantify the downstream impact of plasma mcfDNA sequencing on antimicrobial prescribing behaviors. Third, we excluded participants from this analysis for whom plasma mcfDNA sequencing was not collected or an error occurred during processing. A post hoc analysis tested the null hypothesis that study participants who were excluded from the analysis due to missing plasma mcfDNA sequencing results were missing completely at random. We found no significant differences in observed participant characteristics comparing participants excluded from the analysis with those who were retained. Fourth, plasma mcfDNA sequencing does not detect RNA viruses, and a negative test does not necessarily rule out infection. Moreover, antimicrobial resistance gene markers detected by plasma microbial mcfDNA sequencing may not always correlate with phenotypic resistance. This knowledge is critical when deciding the need for bronchoscopy or narrowing antimicrobial therapy based on plasma mcfDNA sequencing results. For these reasons, our results support a role for plasma microbial cfDNA as a complementary test to usual care testing under most circumstances. Finally, it is important to recognize that plasma microbial test results were unavailable to treating clinicians. Prospective studies that provide detections by plasma mcfDNA sequencing to clinicians in real time may further define the clinical utility of this technique and enable the assessment of important patient-centered outcomes.

In summary, we demonstrate that plasma mcfDNA sequencing could impact clinical decision-making among immunocompromised patients with pneumonia. The use of plasma mcfDNA sequencing in this patient population may be most impactful when applied with initial noninvasive testing or among patients unsuitable for invasive sample collection. When a probable cause of pneumonia is identified by plasma mcfDNA sequencing, the added value of bronchoscopy should be carefully considered. Finally, we observed that the impact of plasma mcfDNA sequencing in this patient population was in part driven by the detection of concurrent nonpneumonia infections in this population with suspected pneumonia. These findings highlight the unique advantage that a hypothesis-free testing approach offers for this patient population.

## Supplementary Material

ofae425_Supplementary_Data

## Supplementary Data

Supplementary materials are available at *Open Forum Infectious Diseases* online. Consisting of data provided by the authors to benefit the reader, the posted materials are not copyedited and are the sole responsibility of the authors, so questions or comments should be addressed to the corresponding author.
