# Supplementary material for: Clinical Utility of Plasma Microbial Cell-Free DNA Sequencing Among Immunocompromised Patients With Pneumonia
Source: Open Forum Infect Dis. 2024 Jul 22;11(8):ofae425. doi: 10.1093/ofid/ofae425 (PMC11292041; doi:10.1093/ofid/ofae425)
Supplement: ofae425_Supplementary_Data [file ofae425_supplementary_data.zip › Supplemental_data_R2.docx]

**Supplemental Content**

**Clinical utility of plasma microbial cell-free DNA sequencing among immunocompromised patients with pneumonia**

Table of Contents

[Section 1: Methods 2](#_Toc171420841)

[Example Plasma Microbial Cell-Free DNA Sequencing Test Report^*^ 2](#_Toc171420842)

[Section 2: Results 7](#_Toc171420843)

[Supplementary Table 1. 7](#_Toc171420844)

[Supplementary Table 2. 8](#_Toc171420845)

[Supplementary Table 3. 9](#_Toc171420846)

[Supplementary Table 4. 10](#_Toc171420847)

[Supplementary Figure 1. 12](#_Toc171420848)

[Supplementary Figure 2. 13](#_Toc171420849)

[Supplementary Figure 3. 14](#_Toc171420850)

[Supplementary Figure 4. 15](#_Toc171420851)

[Supplementary Figure 5. 16](#_Toc171420852)

[Supplementary Figure 6. 17](#_Toc171420853)

[Supplementary Figure 7. 18](#_Toc171420854)

[Supplementary Figure 8. 19](#_Toc171420855)

Section 1: Methods

Example Plasma Microbial Cell-Free DNA Sequencing Test Report^*^
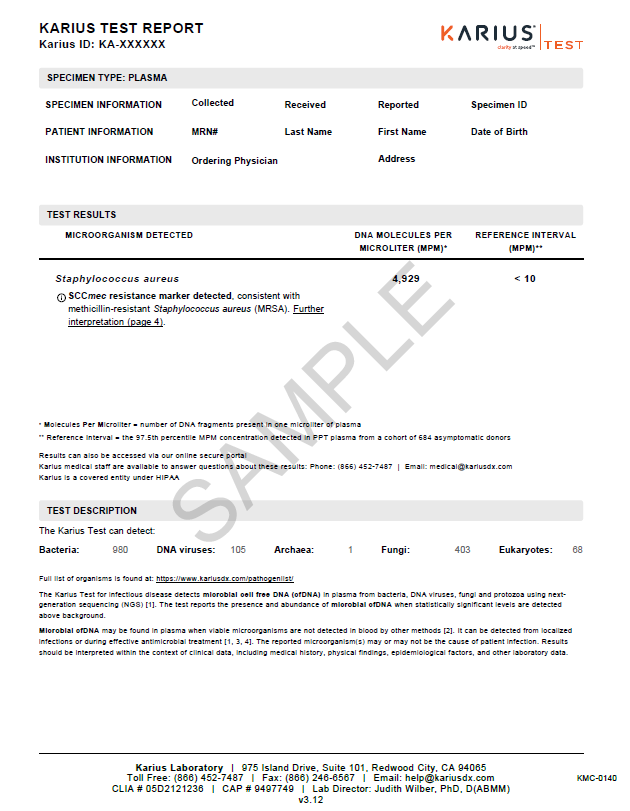


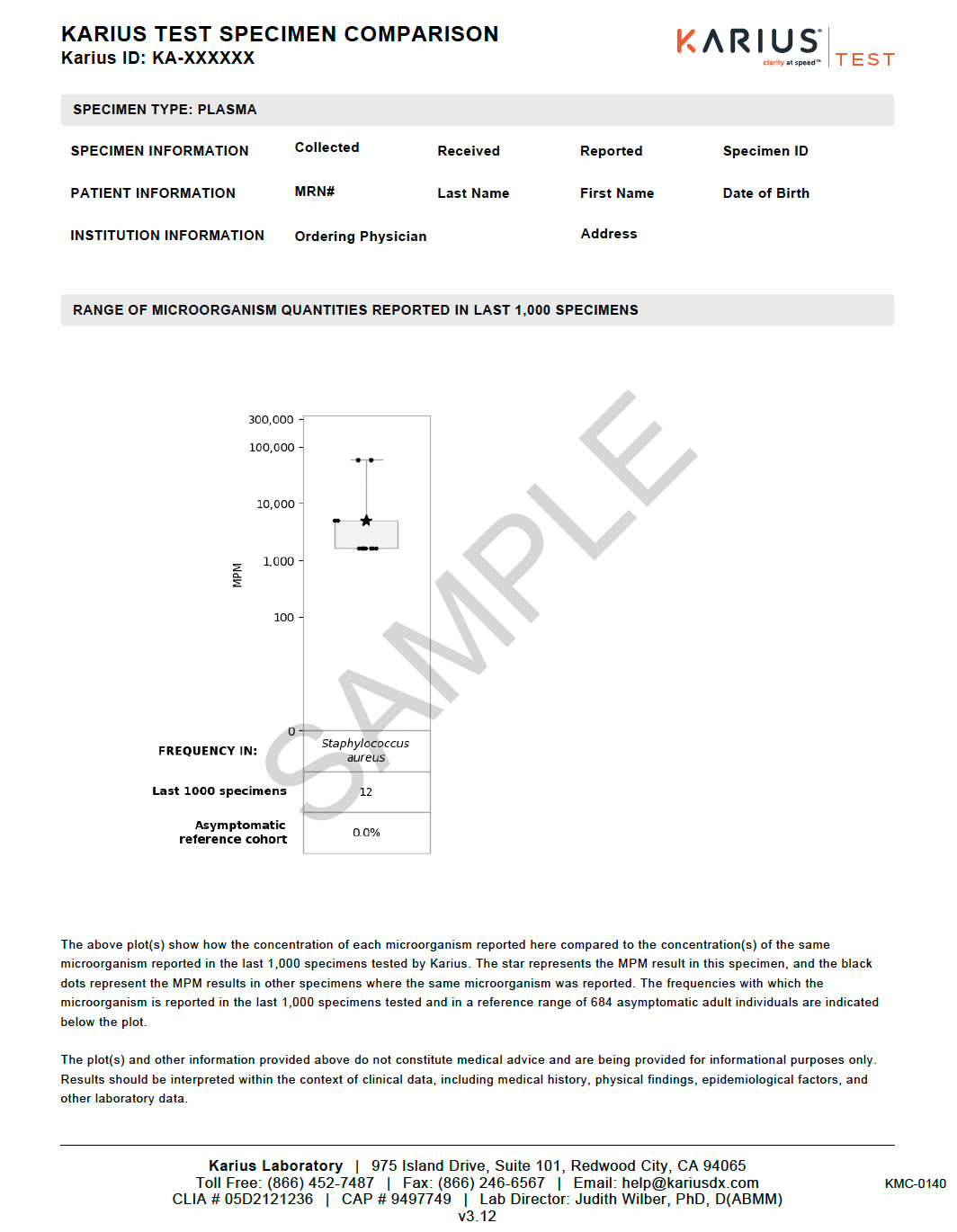


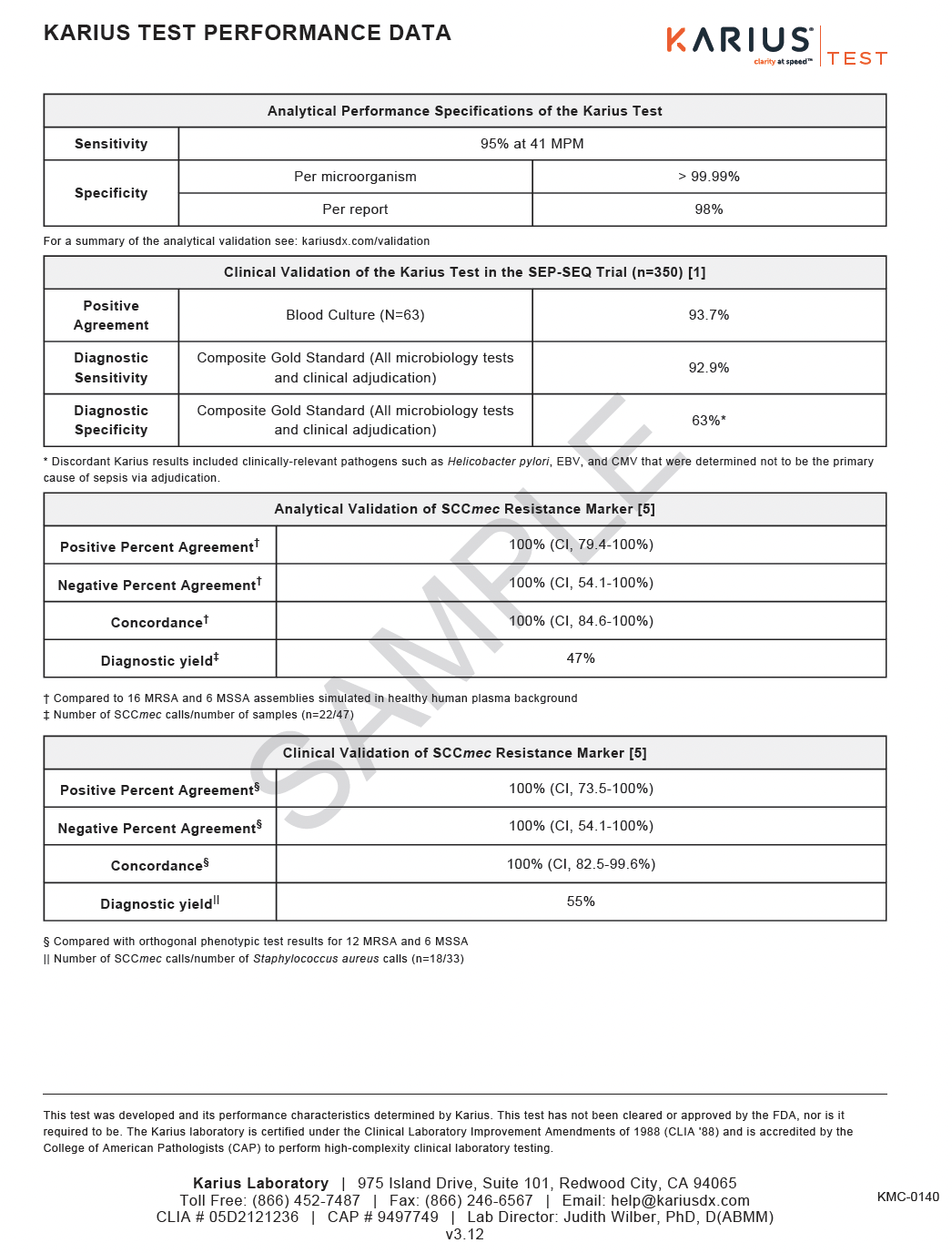


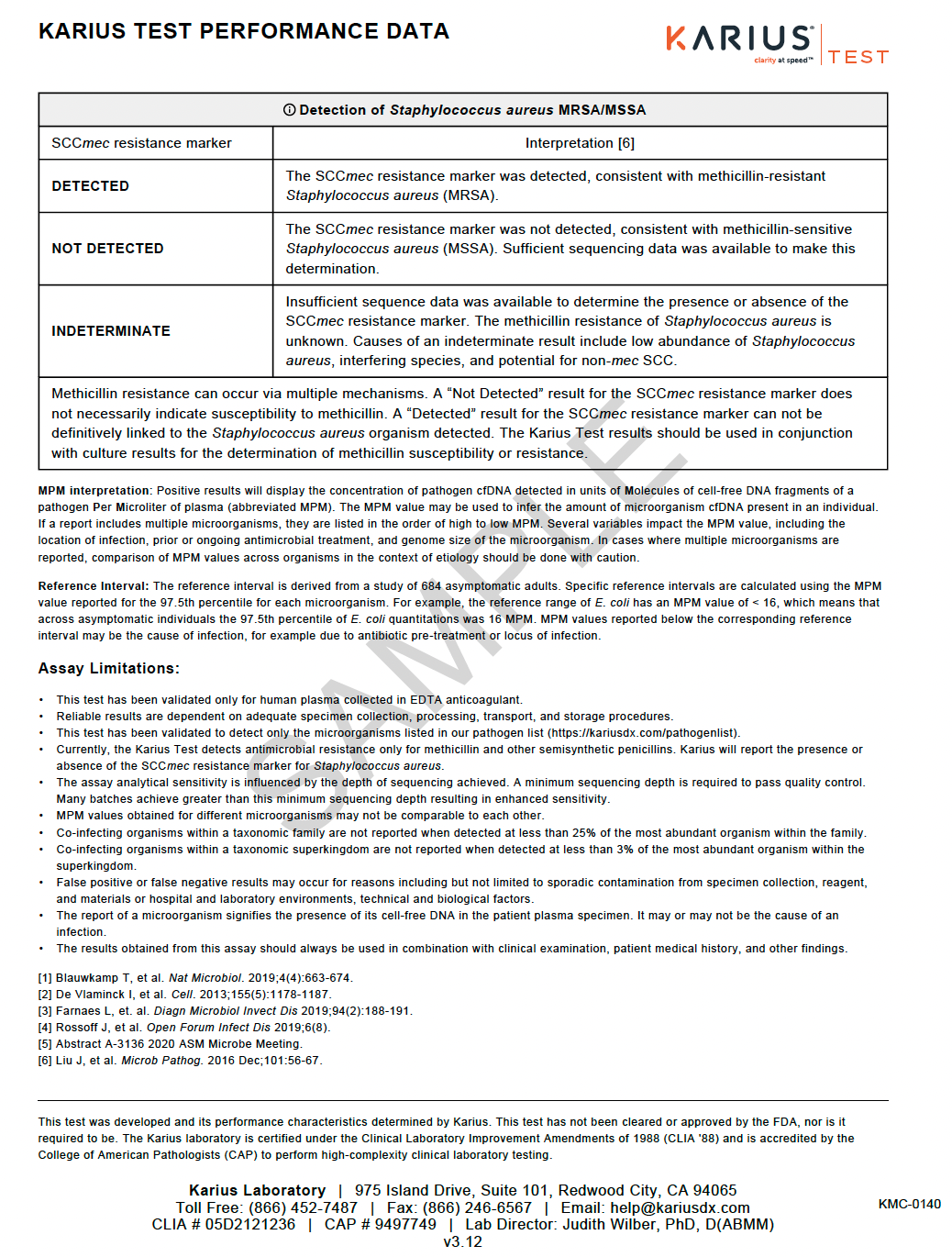


*A sample of the Karius test report available to study adjudicators is presented here. An example of the current clinically available test report, which now includes Antimicrobial Resistance Testing, can be accessed at https://info.kariusdx.com/hubfs/Useful%20PDFs/Sample%20Karius%20Test%20Report.pdf. Antimicrobial resistance gene markers currently available on the Karius test can be accessed at <https://kariusdx.com/karius-test/amr>. The AMR feature of the Karius test was not available during the PICKUP study.

Section 2: Results

Supplementary Table 1. Sputum testing categories and positivity rates among study participants.

| **Sputum Test Type** | **Number of tests** | **Positive (%)** |
| --- | --- | --- |
| Bacterial Culture | 27 | 4 (14.8%) |
| Gram Stain | 19 | 0 (0.0%) |
| AFB Stain | 10 | 0 (0.0%) |
| Fungal Culture | 3 | 0 (0.0%) |
| Fungal Stain | 4 | 0 (0.0%) |
| Other PCR | 3 | 0 (0.0%) |
| PJP Stain | 1 | 0 (0.0%) |
| Total: | 67 | 4 (5.9%) |

Supplementary Table 2. Complications from bronchoscopy among study participants

|  | **Overall (N=223^)** | **Probable cause of PNA identified by plasma microbial cell-free DNA sequencing and missed by Bronchoscopy** **(N=25)** | **Probable cause of PNA identified by both plasma microbial cell-free DNA sequencing and bronchoscopy - same organism** **(N=27)** |
| --- | --- | --- | --- |
| Any complication | 35/222 (15.8%) | 5/25 (20.0%) | 6/27 (22.2%) |
| New/worsened hypoxemia | 18 (8.1%) | 2 (8.0%) | 2 (7.4%) |
| Bronchospasm | 7 (3.2%) | 1 (4.0%) | 0 (0.0%) |
| Cough | 6 (2.7%) | 1 (4.0%) | 1 (3.7%) |
| Respiratory failure/Cardiac arrhythmia/Intubation | 5 (2.3%) | 1 (4.0%) | 1 (3.7%) |
| Airway bleeding | 4 (1.8%) | 0 (0.0%) | 1 (3.7%) |
| Respiratory failure requiring ICU admission/transfer | 3 (1.4%) | 1 (4.0%) | 0 (0.0%) |
| Cardiac arrhythmia requiring treatment | 2 (0.9%) | 0 (0.0%) | 1 (3.7%) |
| Chest pain | 2 (0.9%) | 1 (4.0%) | 1 (3.7%) |
| Shortness of breath | 2 (0.9%) | 0 (0.0%) | 0 (0.0%) |
| Endotracheal intubation | 1 (0.5%) | 0 (0.0%) | 0 (0.0%) |
| Hypotension | 1 (0.5%) | 0 (0.0%) | 0 (0.0%) |
| Procedural bleeding, minor | 1 (0.5%) | 0 (0.0%) | 1 (3.7%) |

^^^One study subject was scheduled for bronchoscopy but did not have the procedure due to declining health status

Supplementary Table 3. Distribution of adjudication categories for plasma microbial cell-free DNA testing

| **Categories** | **Number of patients (%)** |
| --- | --- |
| Only pneumonia | 23 (10.3%) |
| Only clinically relevant non-pulmonary | 53 (23.8%) |
| Only non-clinically relevant | 14 (6.3%) |
| Pneumonia and clinically relevant non-pulmonary | 20 (9%) |
| Pneumonia and non-clinically relevant | 6 (2.7%) |
| Clinically relevant non-pulmonary and non-clinically relevant | 7 (3.1%) |
| Pneumonia, clinically relevant non-pulmonary, and non-clinically relevant | 8 (3.6%) |
| No microbe Identified | 92 (41.3%) |

Supplementary Table 4. Characteristics of study participants per sub-groups defined by changes to antimicrobials for pneumonia and clinically relevant non-pneumonia infections

|  | Plasma microbial cell-free DNA testing changed antimicrobial therapy for pneumonia versus all usual Care microbiologic testing  (N=223) | | Plasma microbial cell-free DNA testing changed antimicrobial therapy for clinically relevant non-pulmonary infections versus all usual Care microbiologic testing  (N=223) | |
| --- | --- | --- | --- | --- |
| **Characteristics** | Yes (N=21) | No (N=202) | Yes (N=22) | No (N=201) |
| Age in years, median (IQR) | 68.0 (50, 71) | 62.0 (50, 69) | 61.5 (55, 68) | 62 (49, 69) |
| Female | 5 (23.8%) | 67 (33.2%) | 5/22 (22.7%) | 67/201 (33.3%) |
| Leukemia | 15 (71.4%) | 131 (64.9%) | 10 (45.5%) | 136 (67.7%) |
| Lymphoma | 4 (19.0%) | 38 (18.8%) | 2 (9.1%) | 40 (19.9%) |
| Myelodysplastic Syndromes (MDS) | 6 (28.6%) | 34 (16.8%) | 6 (27.3%) | 34 (16.9%) |
| Multiple Myeloma (MM) | 0 (0.0%) | 21 (10.4%) | 3 (13.6%) | 18 (9.0%) |
| Transplant | 6/21 (28.6%) | 63/202 (31.2%) | 5/22 (22.7%) | 64/201 (31.8%) |
| Autologous stem cell transplant | 0/6 (0.0%) | 9/63 (14.3%) | 1/5 (20%) | 8/64 (12.5%) |
| Allogenic stem cell transplant | 6/6 (100.0%) | 53/63 (84.1%) | 4/5 (80%) | 47/64 (73.4%) |
| Chemotherapy w/in 45 days of enrollment | 15 (71.4%) | 154 (76.2%) | 15 (68.2%) | 154 (76.6%) |
| Relapse at time of enrollment | 10 (47.6%) | 117 (57.9%) | 13 (59.1%) | 114 (56.7%) |
| Remission at time of enrollment | 5 (23.8%) | 32 (15.8%) | 3 (13.6%) | 34 (16.9%) |
| Active graft versus host disease | 5 (23.8%) | 21 (10.4%) | 5 (22.7%) | 21 (10.4%) |
| Immunosuppressive pharmacologic treatment | 4 (19.0%) | 19 (9.4%) | 4 (18.2%) | 19 (9.5%) |
| Invasive procedure 1 day prior to enrollment to day 14* | 21/21 (100.0%) | 201/202 (99.5%) | 21/22 (95.5%) | 201/201 (100%) |
| Bronchoscopy | 21/21 (100.0%) | 201 (100.0%) | 21 (100%) | 201 (100%) |
| Thoracentesis | 1 (4.8%) | 9 (4.5%) | 0 (0.0%) | 10 (5.0%) |
| Transthoracic needle aspiration | 2 (9.5%) | 1 (0.5%) | 0 (0.0%) | 3 (1.5%) |
| Mini BAL | 0 (0.0%) | 1 (0.5%) | 1 (4.8%) | 0 (0.0%) |
| White blood cell count (K/uL), median (IQR) | 1.10 (0.33, 4.50) | 1.55 (0.50, 4.13) | 0.85 (0.50, 4.50) | 1.60 (0.40, 4.00) |
| Absolute neutrophil count (K/uL), median (IQR) | 1.46 (0.02, 2.43) | 0.64 (0.05, 3.04) | 1.20 (0.03, 3.31) | 0.69 (0.05, 2.92) |
| Anti-infective medication at enrollment | 21/21 (100.0%) | 202/202 (100.0%) | 22/22 (100.0%) | 201 (100.0%) |
| Anti-pseudomonal antibacterial | 21 (100.0%) | 188 (93.1%) | 20 (90.9%) | 189 (94.0%) |
| Mold-active antifungal | 15 (71.4%) | 146 (72.3%) | 17 (77.3%) | 144 (71.6%) |
| Anti-MRSA antibacterial | 16 (76.2%) | 130 (64.4%) | 13 (59.1%) | 133 (66.2%) |
| Anti-PJP antimicrobial | 8 (38.1%) | 46 (22.8%) | 8 (36.4%) | 46 (22.9%) |
| Death <= 30 days | 3 (14.3%) | 34 (16.8%) | 6 (27.3%) | 31 (15.4%) |
| Overall Study Mortality | 3 (14.3%) | 51 (25.2%) | 7 (31.8%) | 47 (23.4%) |

#Data presented as no. (%) unless otherwise indicated

*Includes invasive diagnostic procedures performed to establish pneumonia etiology.

*Some patients underwent ≥1 diagnostic procedure

Abbreviations: MRSA, methicillin-resistant *Staphylococcus aureus*; PJP, *Pneumocystis jiroveci* pneumonia; BAL, bronchoalveolar lavage

Supplementary Figure 1. Flow chart of participants enrolled and included in a secondary analysis of the Pneumonia in the Immunocompromised – Use of the Karius Test for the Detection of Undiagnosed Pathogens (PICKUP) study, 2020-2022

All subjects with an adjudicated plasma microbial cell-free DNA test

(n=223)

**Not adjudicated (n=27)**

No specimen collected and/or no sequencing test result available (n=9)

Withdrawn from study (n=1)

Site sample processing error (n=6)

Shipment error (n=3)

Plasma cell-free DNA testing failure after receipt at Karius lab (n=8)

**Did not meet eligibility criteria (n=7)**

Not immunocompromised (n=3)

Did not have hematologic malignancy (n=2)

Positive for SARS-CoV-2 14 days prior to enrollment (n=2)

Enrolled (n=257)

All eligible enrolled subjects

(n=250)

Supplementary Figure 2. Number of detected taxa counts per report for all positive plasma microbial cell-free DNA sequencing reports
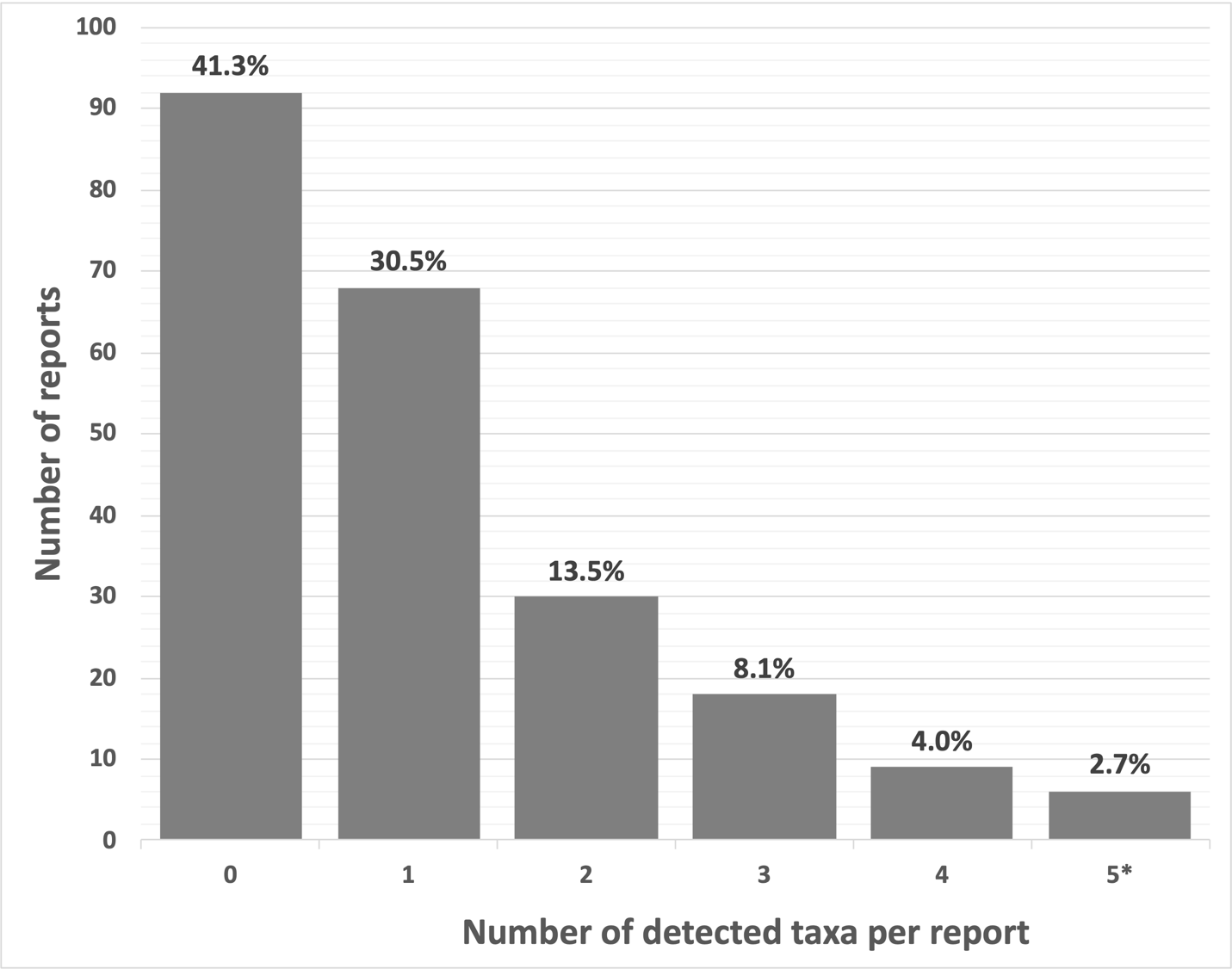


Percentages reflect the proportion of all reports (n = 223)

***** 3 reports had 6 taxa detected each

Supplementary Figure 3. Categorization of 251 pathogens detected by plasma microbial cell-free DNA sequencing


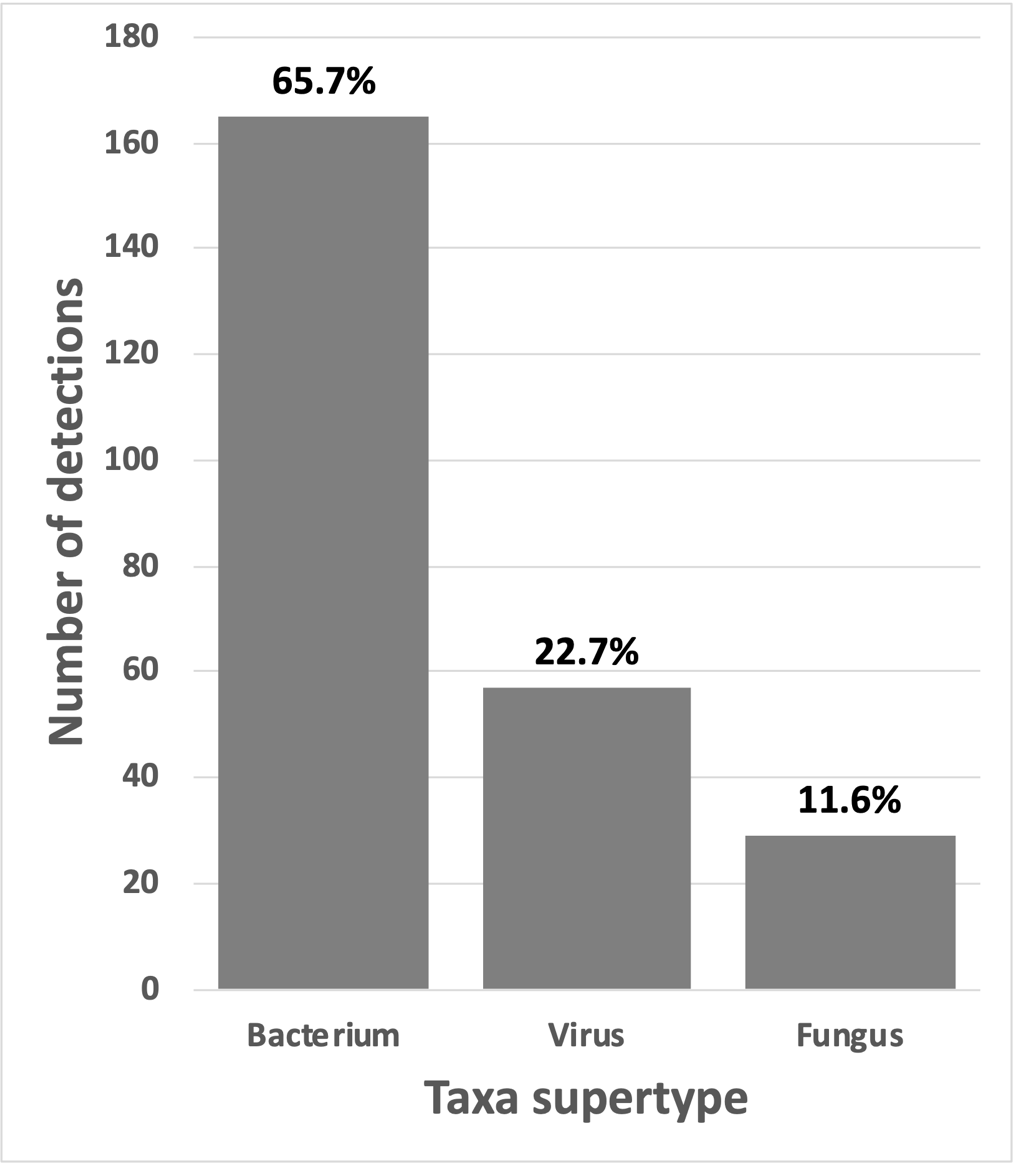


* Percentages reflect the proportion of the total number of detections (bacteria, 165; viruses, 60; and fungi, 26)

Supplementary Figure 4. Distribution of usual care testing and pathogens identified as a probable cause of pneumonia by both plasma microbial cell-free DNA (mcfDNA) sequencing and usual care testing


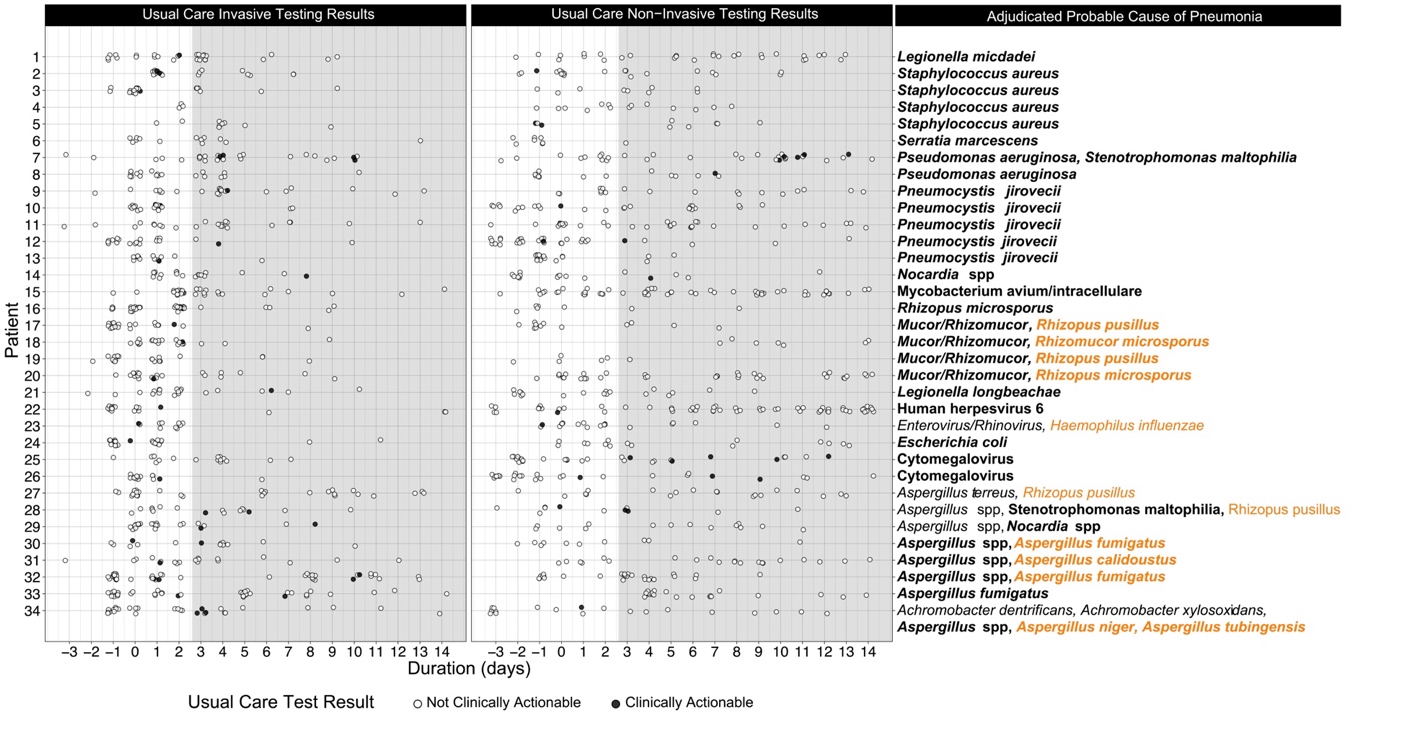


Supplementary Figure 4. Plasma mcfDNA sequencing identified a probable cause of pneumonia in 57 participants. A probable cause of pneumonia was exclusively identified by plasma mcfDNA sequencing in 23 participants and agreed with usual care testing for a probable cause of pneumonia in 34 participants. Probable causes of pneumonia identified among those 34 participants are presented here. Each usual care test performed from -3 to 14 days following enrollment is depicted as a circle and filled black for those that were clinically actionable. The calculated median time from sample collection to results for plasma mcfDNA is 2.63 and this is depicted as the gray-shaded region. Black text – Usual Care only; Orange text – plasma mcfDNA only; Bolded black text – Usual Care and plasma mcfDNA; Orange bold text - Usual Care and plasma mcfDNA, with mcfDNA detecting the spp.

Supplementary Figure 5. Distribution of nondiagnostic usual care testing and pathogens exclusively identified by plasma microbial cell-free DNA sequencing and adjudicated as a probable cause of pneumonia


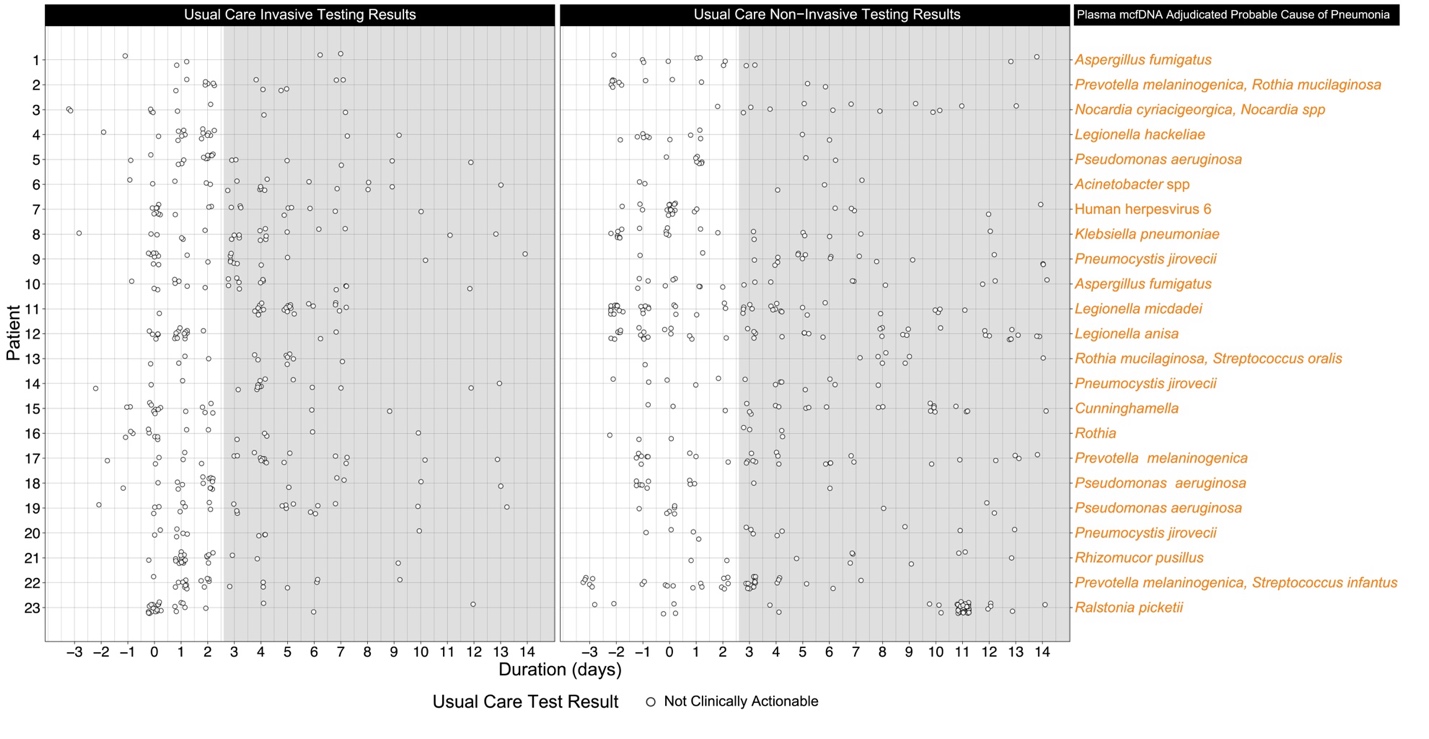


Supplementary Figure 5. Each usual care test performed from -3 to 14 days following enrollment is depicted as a circle. The previously reported median time from sample collection to results for plasma microbial cell-free DNA is 2.63 days and this is depicted as the gray-shaded region.

Supplementary Figure 6. Median time in days to a clinically actionable microbiologic result from specimen collection among participants with an identified probable cause of pneumonia


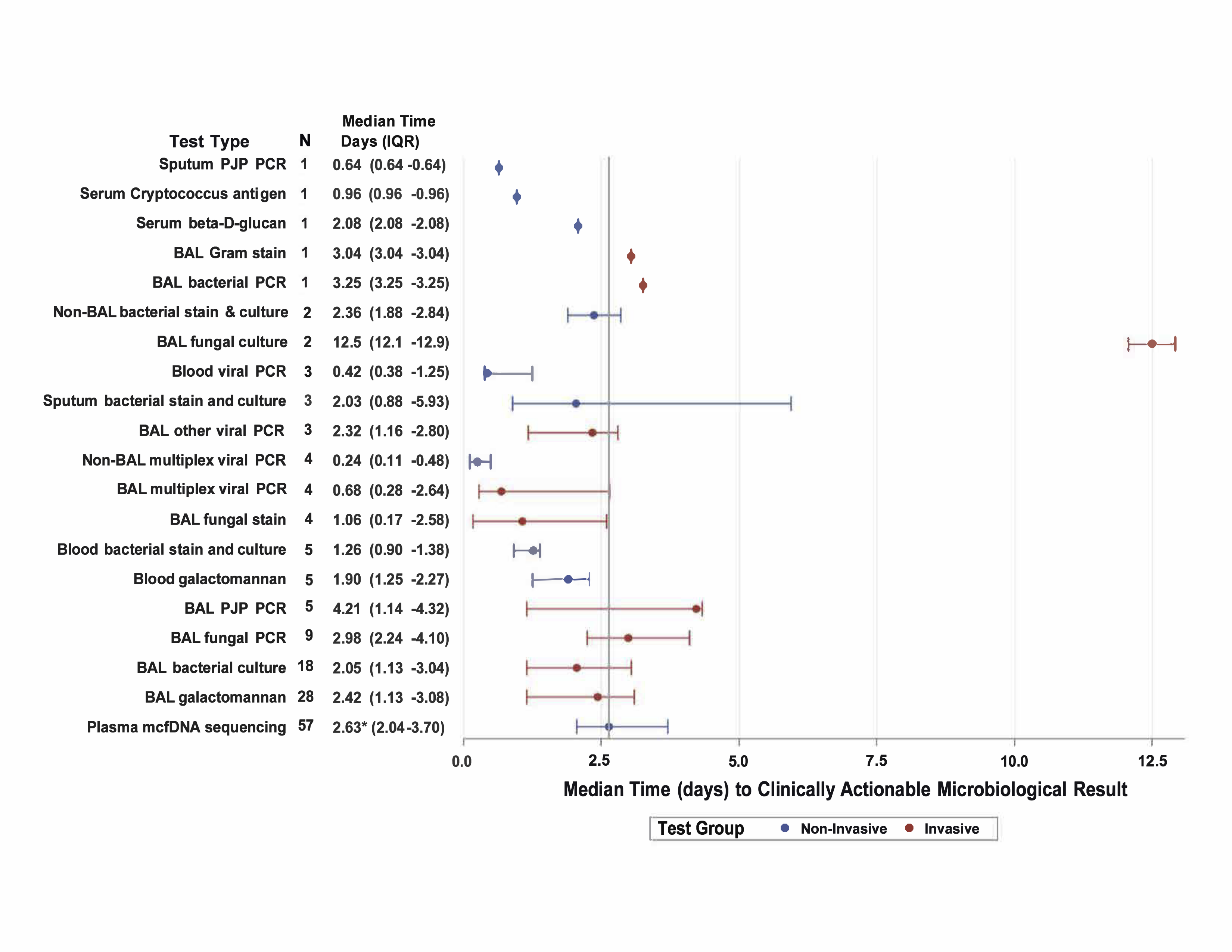


Supplementary Figure 6. N represents the total number of tests with a clinically actionable microbiologic result for the adjudicated probable cause of pneumonia for each test type. Not all participants had tests from each category. Some participants had multiple probable causes of pneumonia identified and multiple tests that identified the same probable cause of pneumonia.

Supplementary Figure 7. Frequency of pathogens among 88 study participants in which plasma microbial cell-free DNA sequencing identified a microbe adjudicated as a cause of a clinically relevant non-pneumonia infection
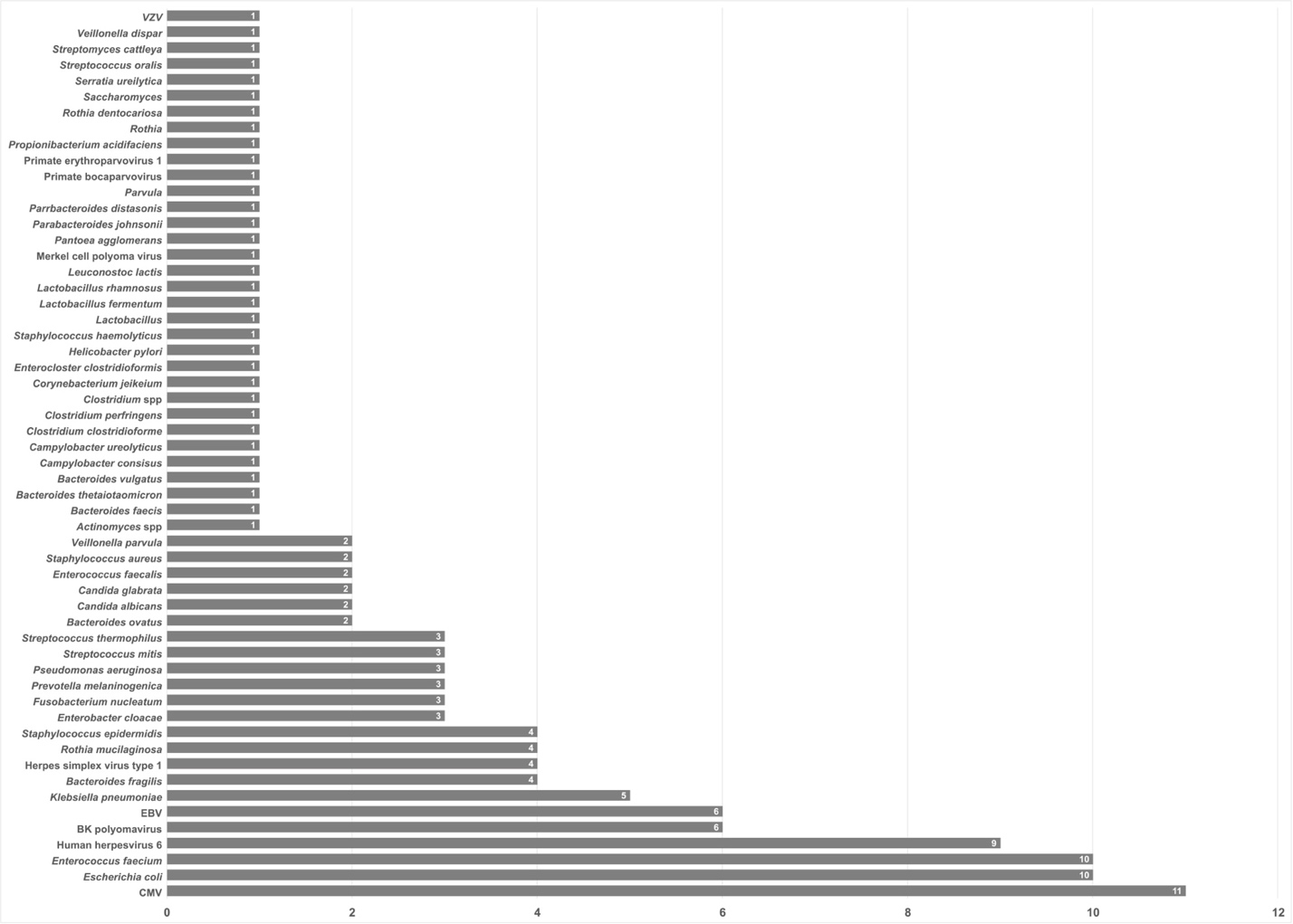


Supplementary Figure 8. Hypothetical placement of plasma microbial cell-free DNA sequencing in the evaluation of immunocompromised patients with pneumonia

**
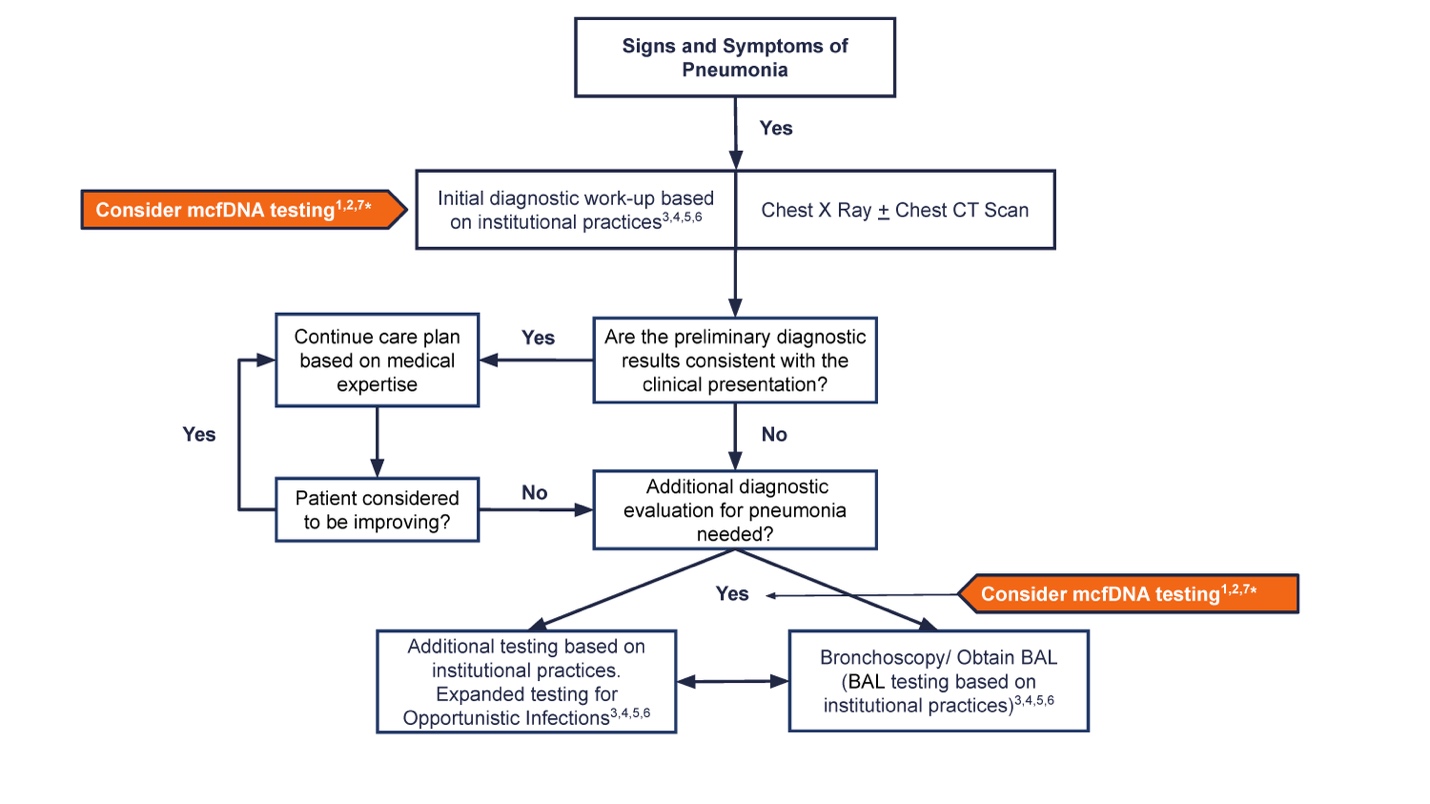
**

**Figure references:**

1. Bergin SP, Chemaly RF, Dadwal SS, et al. Plasma Microbial Cell-Free DNA Sequencing in Immunocompromised Patients with Pneumonia: A Prospective Observational Study. Clinical Infectious Diseases 2023. DOI: 10.1093/cid/ciad599.Foong KS, et al. Open Forum Infect Dis. 2022 Dec 2;9(12):ofac652.
2. Foong KS, et al. Open Forum Infect Dis. 2022 Dec 2;9(12):ofac652
3. Hage CA, Carmona EM, Epelbaum O, et al. Microbiological Laboratory Testing in the Diagnosis of Fungal Infections in Pulmonary and Critical Care Practice. An Official American Thoracic Society Clinical Practice Guideline [published correction appears in Am J Respir Crit Care Med. 2019 Nov 15;200(10):1326]. Am J Respir Crit Care Med. 2019;200(5):535-550. doi:10.1164/rccm.201906-1185ST
4. Miller JM, Binnicker MJ, Campbell S, et al. A Guide to Utilization of the Microbiology Laboratory for Diagnosis of Infectious Diseases: 2018 Update by the Infectious Diseases Society of America and the American Society for Microbiology. Clin Infect Dis. 2018;67(6):e1-e94. doi:10.1093/cid/ciy381
5. Ramirez JA, Musher DM, Evans SE, et al. Treatment of Community-Acquired Pneumonia in Immunocompromised Adults: A Consensus Statement Regarding Initial Strategies. Chest. 2020;158(5):1896-1911. doi:10.1016/j.chest.2020.05.598
6. S.S. Dadwal et al. American Society of Transplantation and Cellular Therapy Series, 2: Management and Prevention of Aspergillosis in Hematopoietic Cell Transplantation Recipients Transplantation and Cellular Therapy 27 (2021) 201-211
7. Vissichelli NC, et al. Transpl Infect Dis. 2023;25(1):e13954.
