# Supplementary figures and images for: Clinical Utility of Plasma Microbial Cell-Free DNA Sequencing Among Immunocompromised Patients With Pneumonia
Source: Open Forum Infect Dis. 2024 Jul 22;11(8):ofae425. doi: 10.1093/ofid/ofae425 (PMC11292041; doi:10.1093/ofid/ofae425)

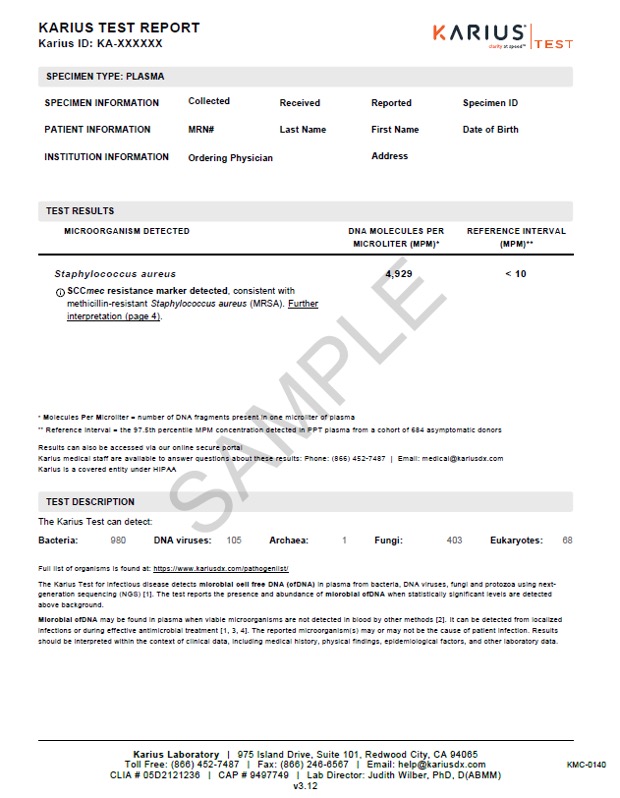

Supplement: ofae425_Supplementary_Data [file ofae425_supplementary_data.zip › KT_test1_methods.jpg]

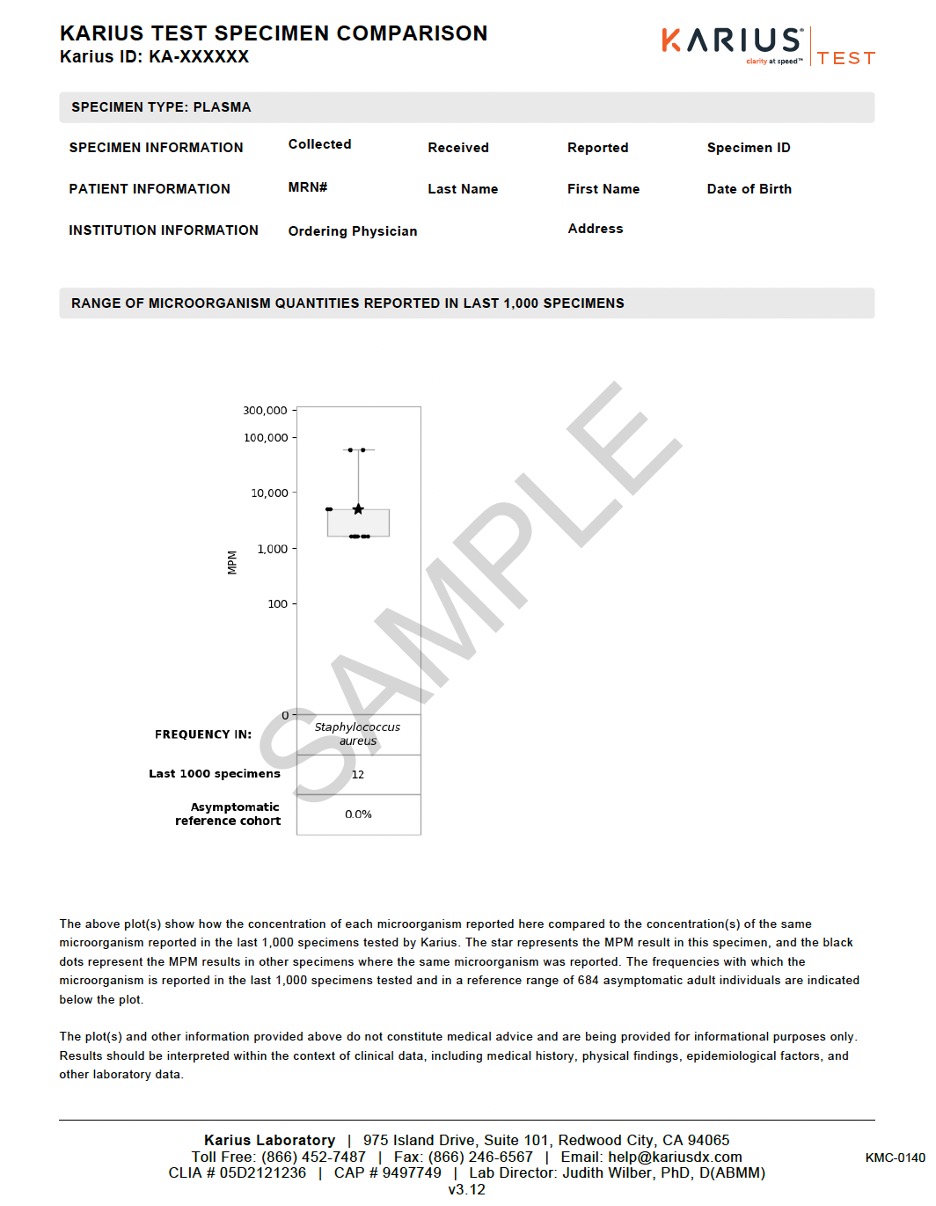

Supplement: ofae425_Supplementary_Data [file ofae425_supplementary_data.zip › KT_test2_methods.jpg]

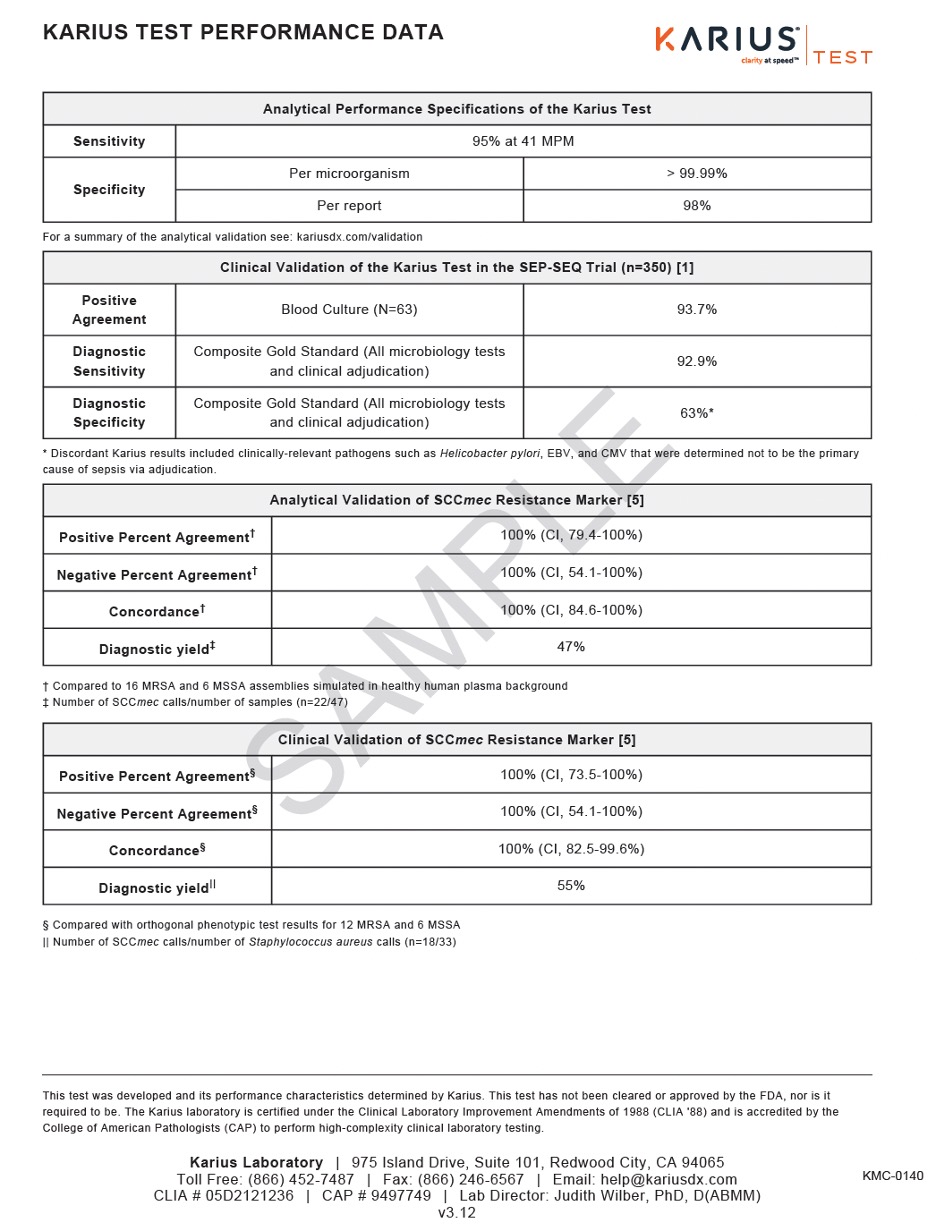

Supplement: ofae425_Supplementary_Data [file ofae425_supplementary_data.zip › KT_test3_methods.jpg]

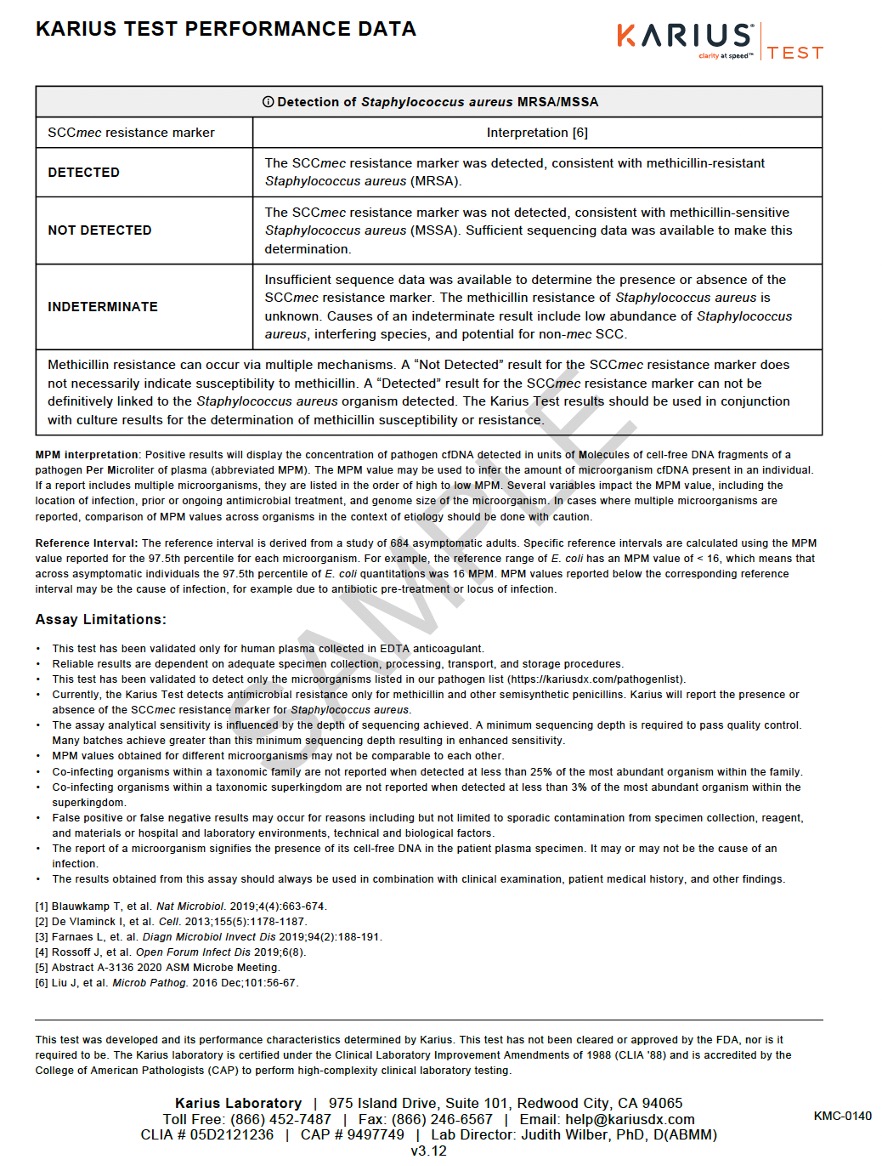

Supplement: ofae425_Supplementary_Data [file ofae425_supplementary_data.zip › KT_test4_methods.jpg]

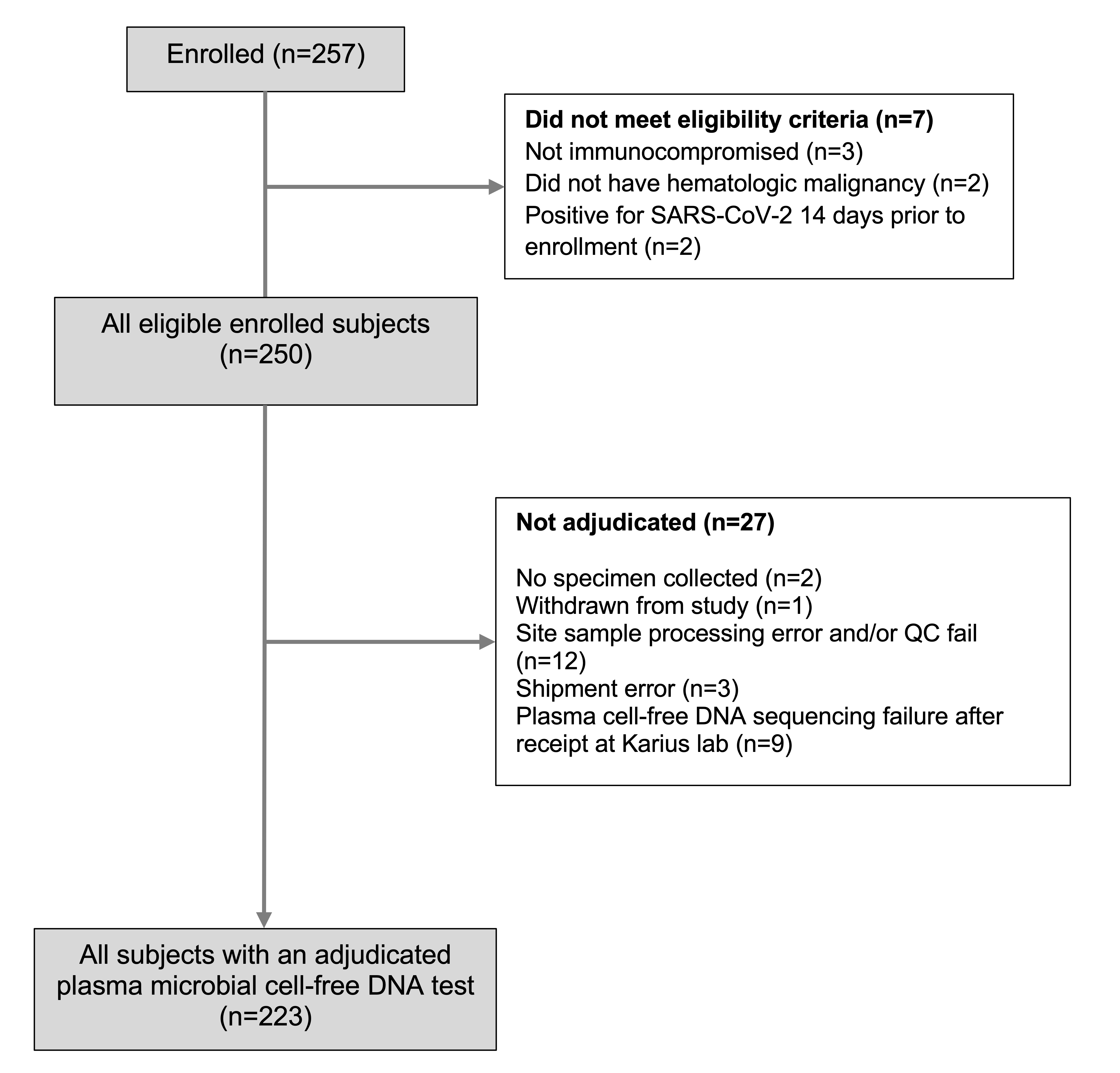

Supplement: ofae425_Supplementary_Data [file ofae425_supplementary_data.zip › SuppFig1.jpg]

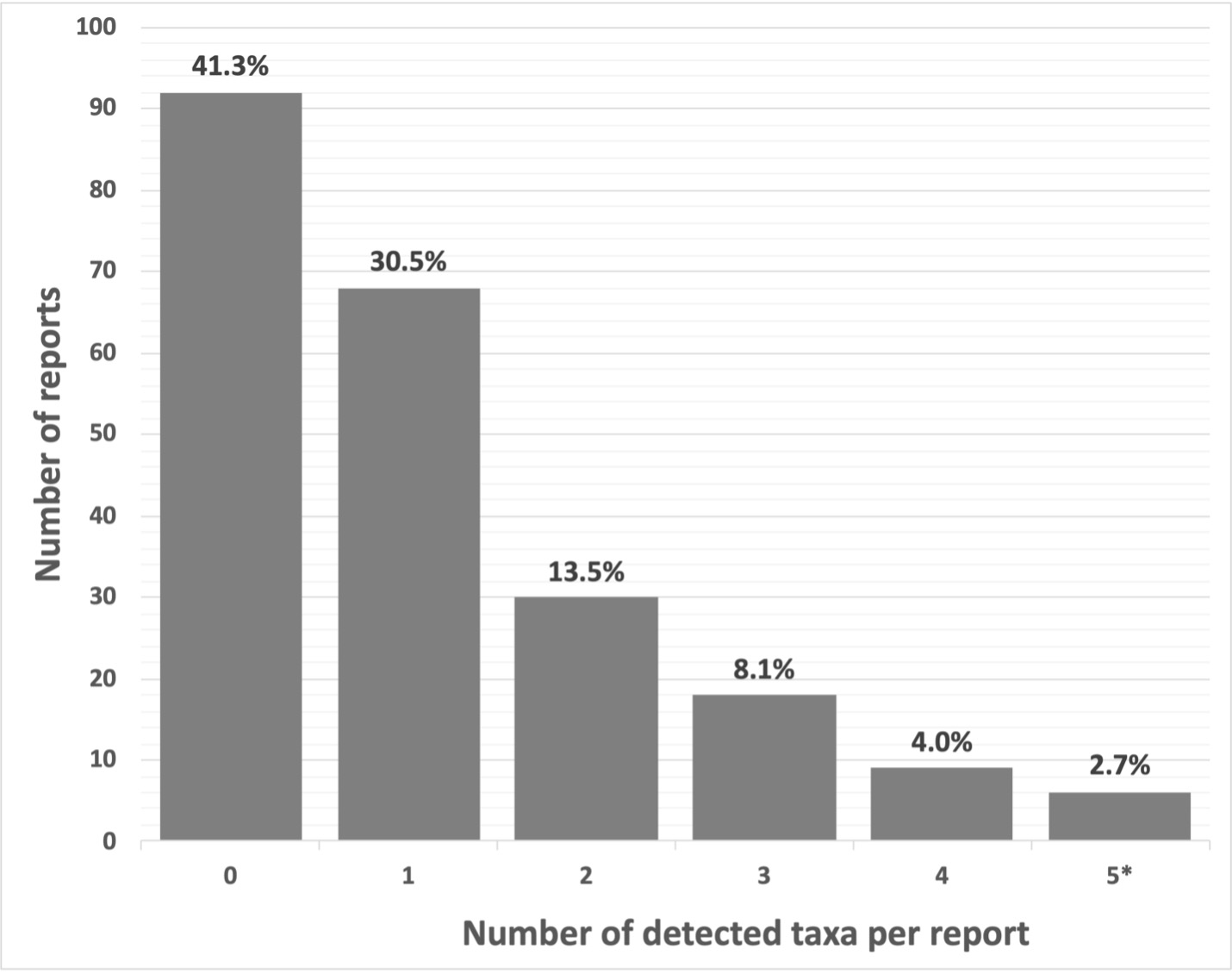

Supplement: ofae425_Supplementary_Data [file ofae425_supplementary_data.zip › SuppFig2.jpg]

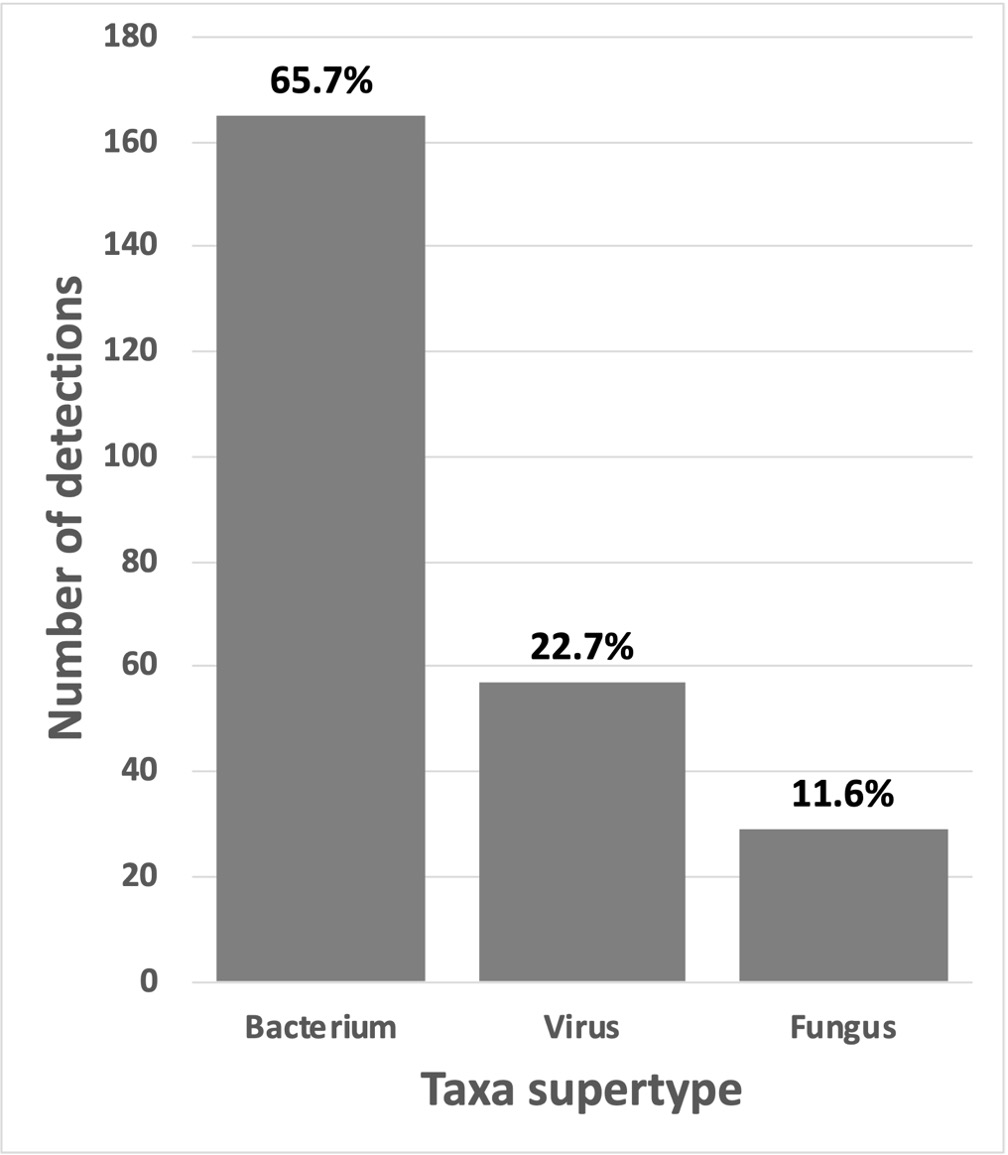

Supplement: ofae425_Supplementary_Data [file ofae425_supplementary_data.zip › SuppFig3.jpg]

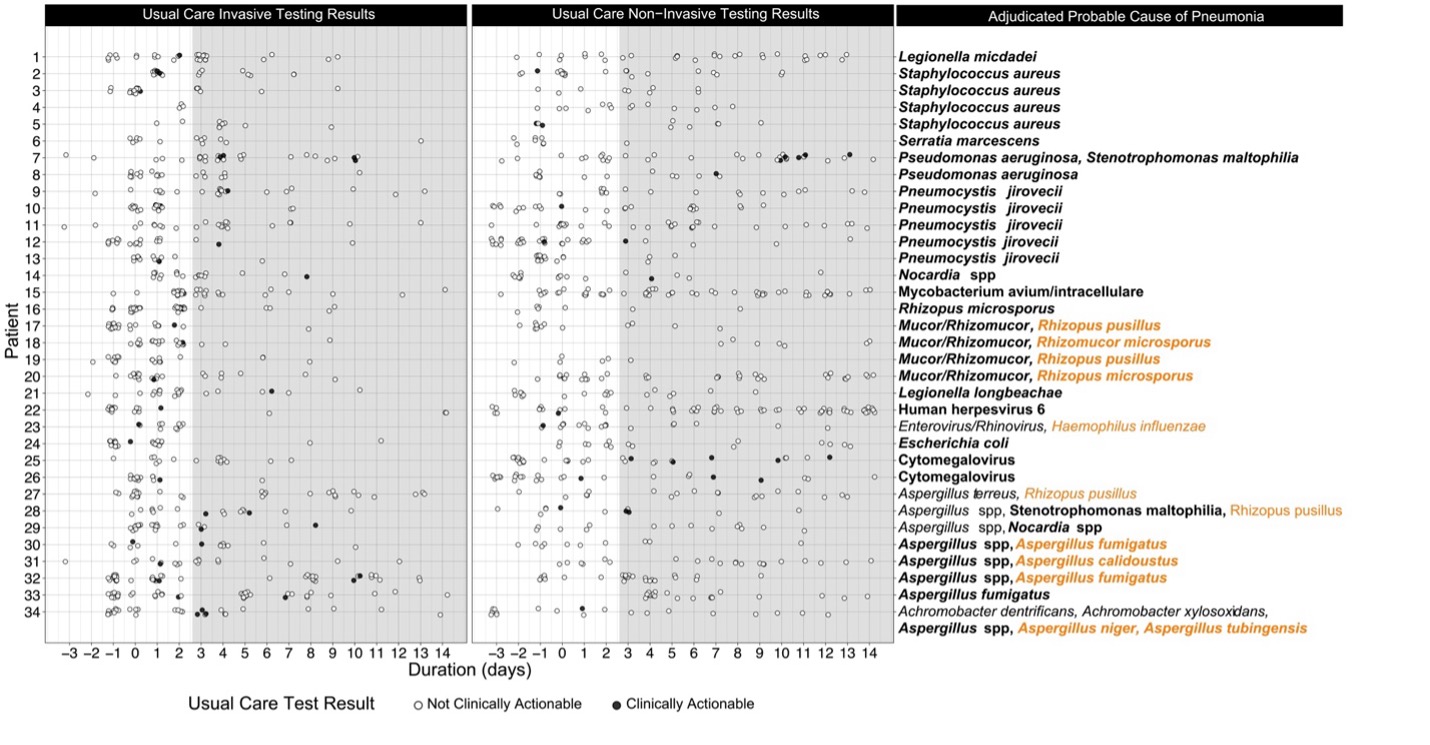

Supplement: ofae425_Supplementary_Data [file ofae425_supplementary_data.zip › SuppFig4.jpg]

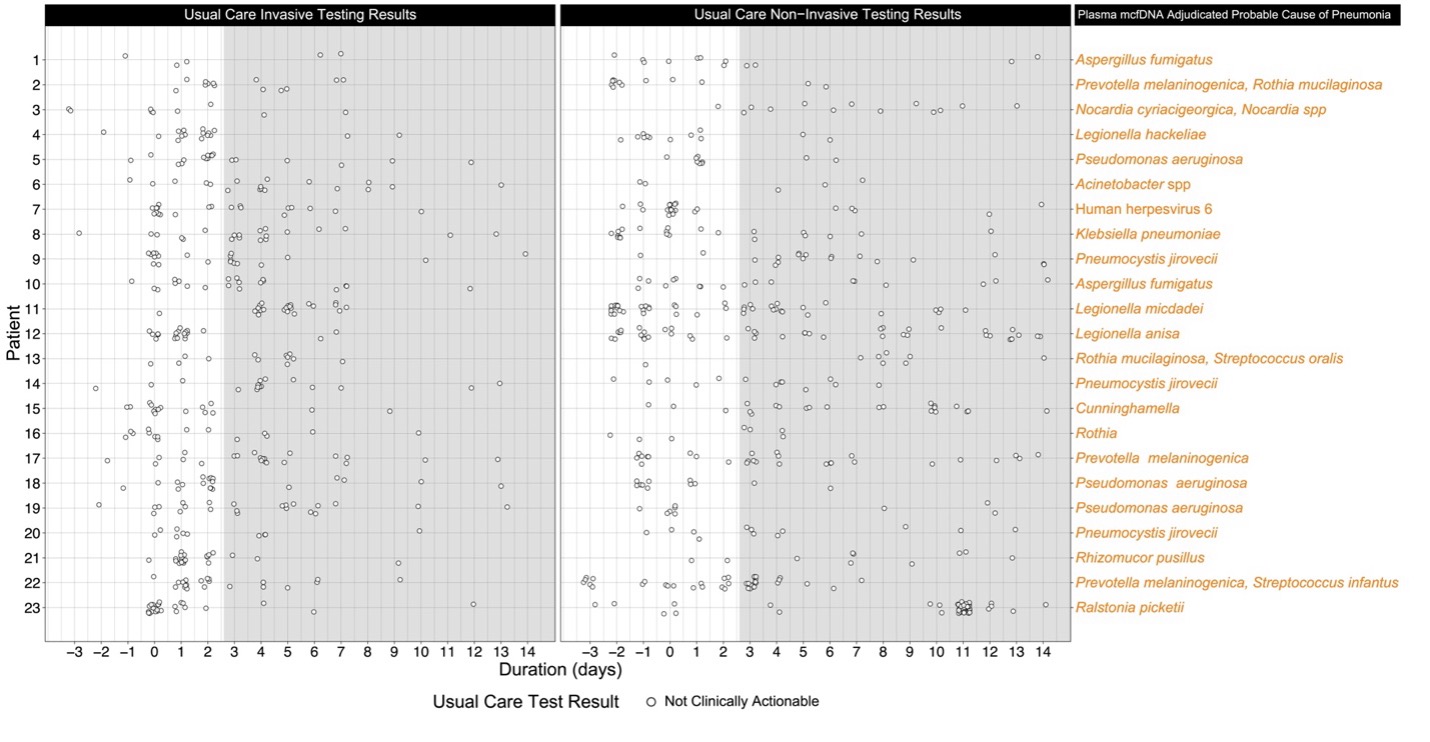

Supplement: ofae425_Supplementary_Data [file ofae425_supplementary_data.zip › SuppFig5.jpg]

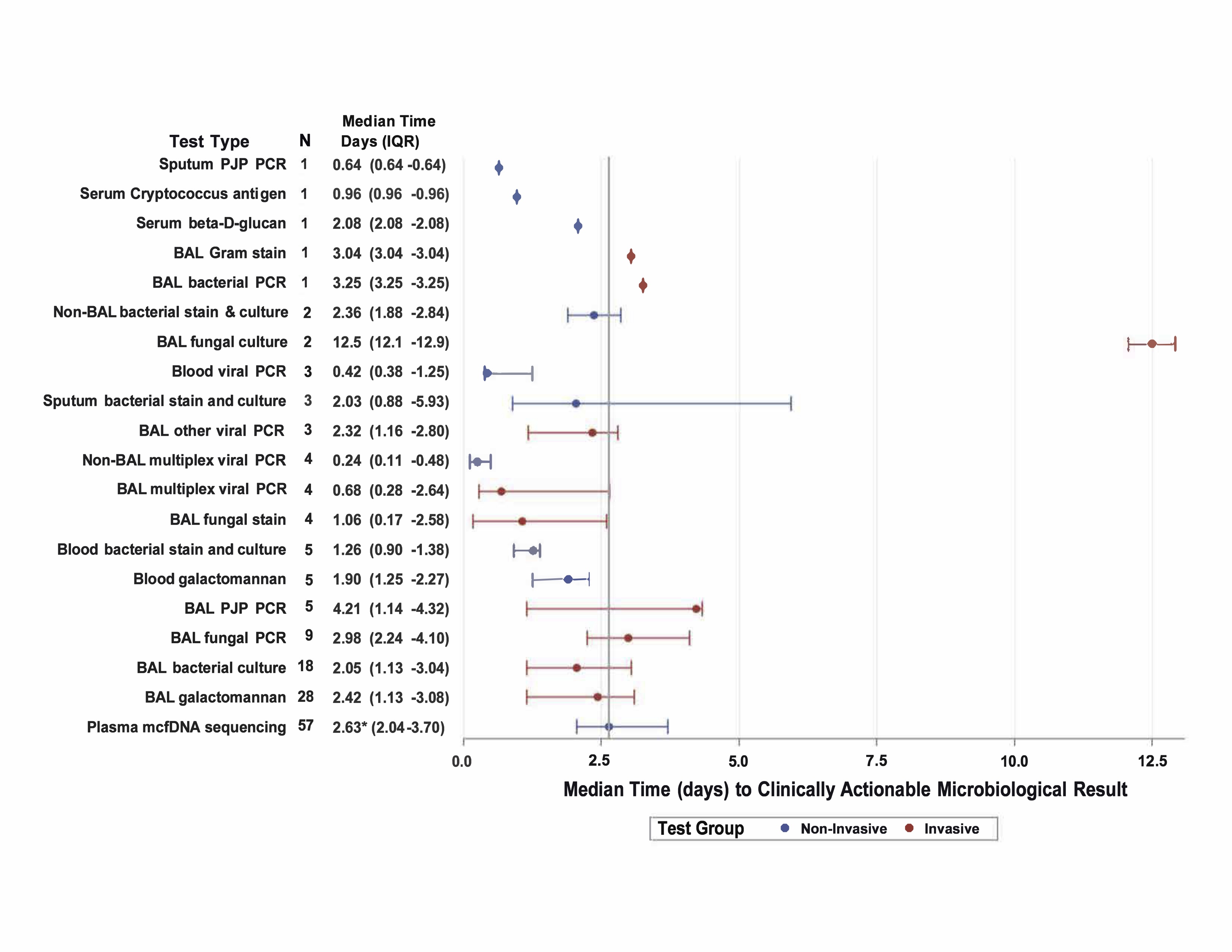

Supplement: ofae425_Supplementary_Data [file ofae425_supplementary_data.zip › SuppFig6.jpg]

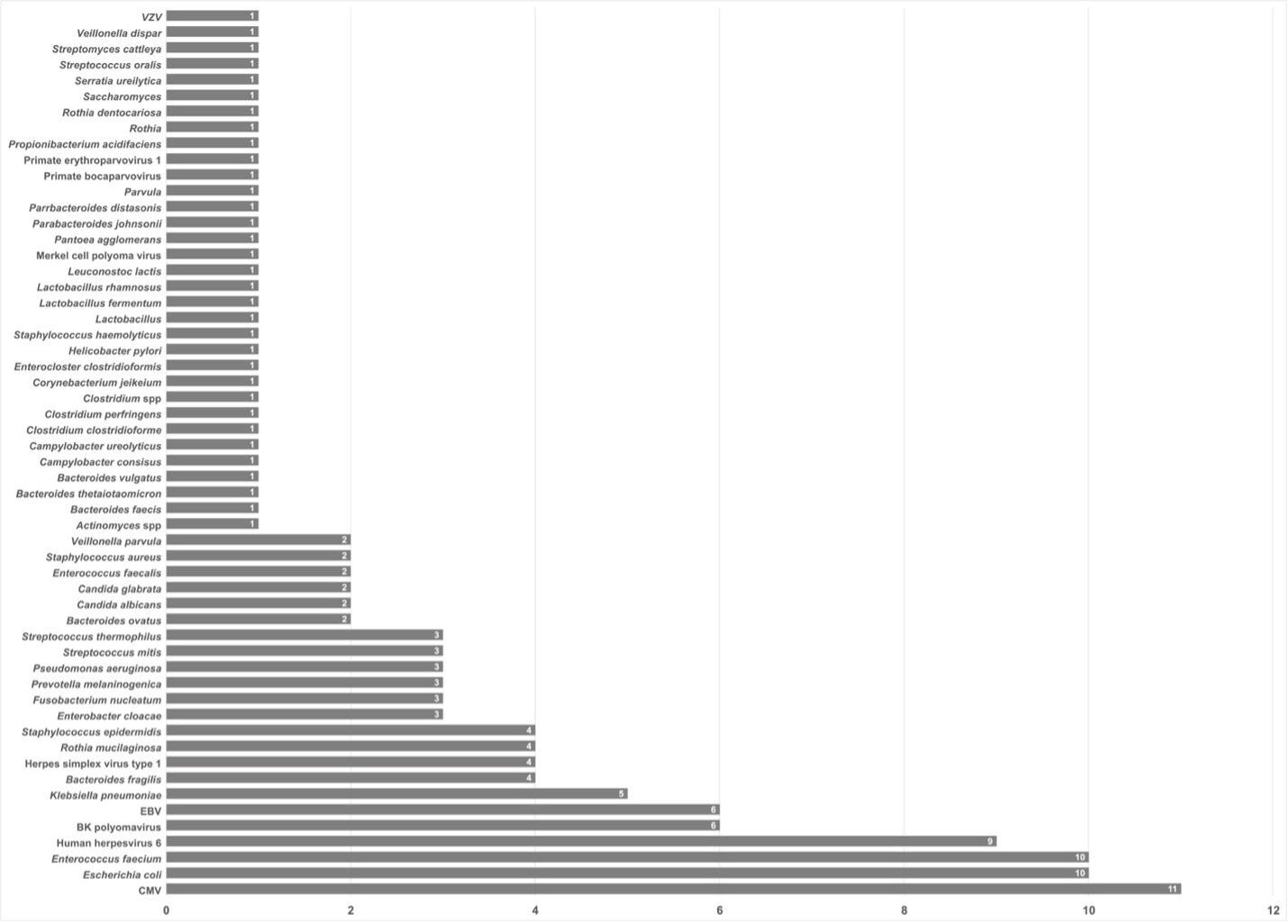

Supplement: ofae425_Supplementary_Data [file ofae425_supplementary_data.zip › SuppFig7.jpg]

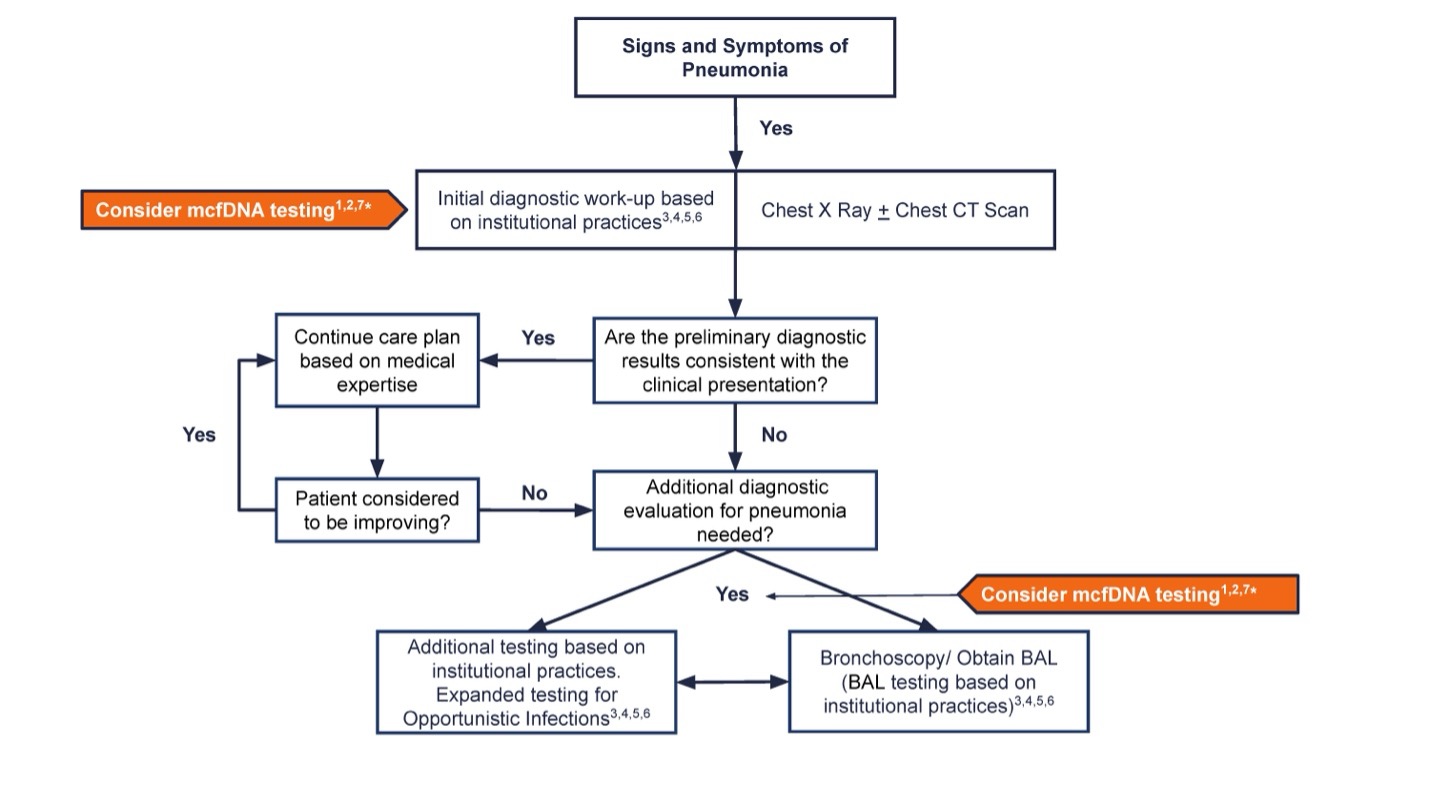

Supplement: ofae425_Supplementary_Data [file ofae425_supplementary_data.zip › SuppFig8.jpg]
